# Effect and mechanism of apelin on lipopolysaccharide induced acute pulmonary vascular endothelial barrier dysfunction

**DOI:** 10.1038/s41598-023-27889-6

**Published:** 2023-01-27

**Authors:** Tianpeng Huang, Danyang Chen, Wei Ye, Wenwen Chen, Min Zhang, Jiale Hao, Licong Xu, Xiaoqing Bai, Sunzhong Mao

**Affiliations:** 1 Zhejiang Chinese Medical University Affiliated Wenzhou Hospital of Traditional Chinese Medicine, Wenzhou Hospital of Traditional Chinese Medicine, Wenzhou, 325000 China; 2 School of Basic Medical Sciences, Wenzhou Medical University, Wenzhou, 325035 China; 3 The First People’s Hospital of Pingdingshan, Pingdingshan, 467002 China; 4 Reproductive Medicine Center, Quzhou People’s Hospital, Quzhou, 324000 China; 5 Department of Pathology, School of Basic Medical Sciences, Wenzhou Medical University, Wenzhou, 325035 China

**Keywords:** Infection, Cell biology, Drug discovery, Diseases, Respiratory tract diseases

## Abstract

Vascular endothelial barrier dysfunction is the most prominent manifestation and important cause of mortality in infectious acute lung injury (ALI). Exogenous apelin is effective in ameliorating lipopolysaccharide (LPS)-induced inflammatory response in ALI lungs, reducing exudation of lung tissue and decreasing mortality. This study set out to investigate the association between apelin and Friend leukemia integration-1 (Fli-1) in the prevention and treatment of ALI, and to elucidate the molecular mechanism by which apelin protects the permeability of the vascular endothelial barrier. At the vivo functional level, lung wet/dry weight ratio was used to detect whole lung permeability, evans blue assay and dual fluorescent protein tracking assay were used to detect lung vascular endothelial permeability, HE staining to observe the inflammatory status of lung tissue, and immunofluorescence staining for VE-cadherin expression levels in blood vessels. The changes in inflammatory factors in bronchoalveolar lavage fluid (BALF) were detected by ELASA. Western blot was used to detect the expression level of proteins. qRT-PCR was performed to detect changes in mRNA expression of Fli-1 and adherent junction-related proteins. The correlation analysis of Fli-1 with vascular endothelial permeability and SRC showed that Fli-1 participated in the process of ALI. After preventive and therapeutic treatment of ALI mice with exogenous apelin, Fli-1, APJ, VE-cadherin, phosphorylated-VE-cadherin (p-VE-cadherin) and β-catenin were up-regulated, while SRC, phosphorylated-SRC (p-SRC), VEGF and VEGF-R were down-regulated, which indicated that the stability of vascular endothelial barrier was enhanced. With the use of Fli-1 inhibitor irinotecan, the protective effect of apelin was weakened in various functional indexes, genes and proteins. The lung was maintained at the level of the injury. Our research shows that Fli-1 is involved in the LPS-induced ALI process. The molecular mechanism for apelin in preventing endothelial barrier dysfunction in ALI is through up-regulating Fli-1, thus regulating adherens junction-related proteins, and finally recovering the endothelial barrier function.

## Introduction

Infectious ALI caused by exogenous pathogens is the most common respiratory disease. The pathogenesis of lung injury in infectious ALI includes surface active dysfunction, damage to the epithelium of the alveolar barrier associated with the pulmonary vascular endothelium and activation of the innate immune response. Of particular importance is the damage to the vascular endothelial barrier in the course of ALI. The disruption of the vascular endothelial barrier leads to increased vascular permeability. A large amounts of fluid, inflammatory cells and inflammatory factors permeate into the interstitial tissues of lung, causing edema of lung tissue and aggravating lung.

The pulmonary vascular endothelial barrier is mainly composed of adherens junction and tight junction^1^. The VE-cadherin-catenin complex is the biochemical basis for the formation of adherens junctions between endothelial cells^2^. Once the VE-cadherin-catenin complex is disrupted, it will cause cytoskeletal rearrangement and disrupt the adherens junctions between endothelial cells. This will result in dysfunction of endothelial barrier and increases endothelial permeability^3,4^. Endotoxins have been found to disrupt endothelial barrier function and stimulate vascular permeability such as LPS^5^. During the process of sepsis-induced ALI, LPS triggers endothelial barrier dysfunction by activating TLR4 receptors. However, the signal transduction mechanism of LPS-induced decrease of endothelial barrier function and increase of permeability has not been fully clarified.

Fli-1 is a transcription factor member of the ETS gene family, and it is mainly found in vascular endothelial cells, hematopoietic systems, immune cells and fibroblasts. A study found that Fli-1 regulates the expression of genes involved in the maintenance of vascular homeostasis, such as VEGF, platelet endothelial cell adhesion factor-1 (PECAM-1), VE-cadherin and S1P^6,7^. This suggests that the Fli-1 mediated endothelial cell homeostatic system plays an important regulatory role in vascular endothelial homeostasis, vascular skeleton formation and angiogenesis. However, it is unclear whether the Fli-1 mediated endothelial cell homeostatic system is involved in the process of pulmonary microvascular endothelial barrier dysfunction caused by infectious ALI.

Improving the endothelial barrier is a key component of ALI repair. Apelin, a small peptide, is a natural ligand for angiotensin II type 1 receptor-related protein (APJ). Studies have shown that apelin has a wide range of physiological effects, with functions such as regulation of circulatory homeostasis, pro-endothelial proliferation and angiogenesis^8^. Previous studies have confirmed that apelin/APJ system is an endogenous anti-injury system, which fights ALI through its anti-inflammatory mechanism^9^. However, little is known about the molecular mechanisms.

The function of apelin in endothelial cells has received much attention in recent years. Kidoya et al. found that apelin inhibited VE-cadherin internalization by upregulating VE-cadherin and p120 protein expression, inhibited VE-cadherin internalization and improved VEGF-mediated elevated vascular permeability^10^. This indicates that apelin is involved in maintaining functional homeostasis in endothelial cells. Besides, it has been demonstrated that the DNA binding recognition sites of Fli-1 and other ETS factors are mainly located at the promoters of blood and endothelial development regulatory factors. After co-knockdown of Erg and Fli-1, the results showed that the genes regulating cell adhesion and endothelial homeostasis were significantly altered, including fatty acid binding protein 4, lymphatic vessel endothelial hyaluronanLYVE-1 and apelin-encoding gene APLN^11^. It is suggested that there may be some association between apelin and the Fli-1-mediated vascular endothelial cell homeostatic system. Thus, it is necessary to further elucidate the role of apelin in preventing ALI in relation to the Fli-1-mediated endothelial cell homeostasis system. In this experiment, we investigated whether Fli-1 is involved in the dysregulation of the pulmonary vascular barrier in ALI. Furthermore, whether the effect of apelin against ALI is related to the Fli-1 mediated signaling pathway in the LPS-induced ALI mouse model.

## Materials and methods

### Animal models

Adult male ICR mice, weighing 30–35 g, were used for the experiments. Drug doses: LPS: 7.5 mg/kg, apelin: 1 mg/kg·d, apelin was injected every 8 h, irinotecan hydrochloride: 1 mg/kg. All drugs were administered by saline dissolution and intraperitoneal injection. All animal experiments were approved by the Institutional Animal Care and Use Committee of Wenzhou Medical University (Wenzhou, China). It also complies with the ARRIVE guidelines and was carried out in accordance with the U.K. Animals (Scientific Procedures) Act, 1986 and associated guidelines.

### Lung wet/dry weight ratio

Whole mouse lungs were taken, and the surface was washed in physiological saline. Filter paper was used to absorb the residual moisture on the lung surface. The wet weight of lungs was measured with electronic balance. Each lung was placed in an oven at 65 °C, and the lungs were removed after 24 h. The dry weight of the lungs was measured. Finally, the lung wet/dry weight ratio was calculated.

### Evans blue assay for pulmonary vascular endothelial permeability

Evans blue was dissolved in sterilized saline, containing 100u of heparin per milliliter, and filtered. Evans blue was injected into the tail vein at a dose of 20 mg/kg. 30 min later, mice were anesthetized with sodium pentobarbital and the lungs were perfused with heparin saline. Whole lungs were taken, dried and weighed for dry weight. The lungs were mixed with PBS, PBS volume (μl) = lung dry weight (mg) × 3.5, and ground into homogenates. Then, a certain amount of formamide was added in the ratio of homogenate to formamide volume of 2:1, incubated at 60 °C for 12–18 h, centrifuged at 2000*g* for 30 min, and the supernatant was collected. 620 nm and 740 nm fluorescence intensity of the supernatant was measured, and the lower absorbance at 740 nm indicated the lower heme contamination. The ratio of the 620 nm fluorescence intensity of the supernatant to the dry weight (mg) of the lung was used to evaluate pulmonary vascular endothelial permeability.

### Dual fluorescent protein tracking assay for pulmonary vascular endothelial permeability

Mice were anesthetized and the jugular sinus was exposed with scissors. 5 μl FITC-BSA was injected via the jugular vein. After 2 h, 4 μl TRITC-BSA was given via the jugular vein. 2 min later, blood was taken from the abdominal aorta and placed away from light. Heparin saline was perfused and rinsed, then lung tissue was taken and weighed. Each group prepared homogenate with equal amount of saline, centrifuged at 2000*g* for 5 min. The supernatant was taken and placed away from light. The fluorescence intensity of FITC (Ex: 490–495 nm, Em: 520–530 nm) and TRITC (Ex: 547 nm, Em: 572 nm) in the plasma and supernatant were measured. Vascular endothelial permeability (U/mg) = [(lung tissue supernatant FITC/plasma FITC) − (lung tissue supernatant TRITC/plasma TRITC)]/tissue weight (mg).

### Bronchoalveolar lavage fluid inflammatory factor assay

Mice were killed with inhalation anesthesia. The whole lung was taken, the syringe needle was tied in the trachea and gently rinsed three times with 0.9 ml of PBS. BALF was collected into a 1.5 ml tube and the supernatant was removed by centrifugation at 300*g* for 10 min. The levels of each inflammatory factor were measured using enzyme-linked immunosorbent assay (ELASA).

### Western blot

Protein samples were obtained by homogenization of mouse lung tissues with high performance RIPA lysate and protease inhibitor, and the supernatant was centrifuged at 4 °C. The total protein content of each group of samples was determined by BCA assay. 100–150 μg of total protein was used as the loading volume. Electrophoresis was performed using 5% upper layer gel and 10% lower layer gel, and the transfer conditions were constant current 300 mA for 90 min, using 0.45 μm PVDF membrane as solid phase carrier. The protein bands were visualized by chemiluminescence.

### Quantitative Real-time PCR

Total RNA of the samples was extracted using Trizol and reverse transcribed into cDNA. The expression of mRNA of each gene was detected by qRT-PCR. qRT-PCR was performed using the SYBR Green.

### Hematoxylin–eosin staining

Mice lungs were sequentially perfused with heparin saline and 4% paraformaldehyde (PFA), and then the lungs were fixed by immersion in 4% PFA for 10 h. The lungs were embedded with paraffin and sectioned. Paraffin sections were dewaxed with xylene and graded concentrations of ethanol. Hematoxylin stained the nuclei and eosin stained the cytoplasm. Xylene and gradient ethanol were used to dehydrate the tissue to transparency. Finally, the sections were sealed with neutral gum.

### Histological analysis

Lung injury was quantified by the ATS Lung Injury Scoring System: (A) neutrophils in the alveolar space, (B) neutrophils in the interstitial space, (C) hyaline membrane formation, (D) intraalveolar proteinaceous material, and (E) alveolar septal thickening; score = [(20 × A) + (14 × B) + (7 × C) + (7 × D) + (2 × E)]/(number of 100 × fields)^12^.

### Immunofluorescence staining

Paraffin sections were baked in a 60 °C incubator for 60 ~ 120 min. Slices were deparaffinized and rehydrated using a series of washes in xylene (× 3) and graded alcohol solutions (100% ethanol × 2, 95% ethanol × 1, and 70% ethanol × 1), followed by washing in water and PBS. Antigen repair: sections were placed in sodium citrate buffer at 100 °C for 30 min and washed in water and PBS. 0.1% triton was punched for 15 min and washed twice in PBS. 3% H_2_O_2_ for 10 min at room temperature, 2 washes in PBS. Non-immune animal serum was incubated at room temperature for 20 min, specific primary antibody was added for 10 min at room temperature and overnight at 4 °C. The next day, re-warm at room temperature for 20 min, wash 3 times with PBS. The fluorescent secondary antibody was added dropwise, protected from light for 1 h at room temperature, and washed 3 times with PBS. All washing was carried out for 5 min. Finally, the mounting medium was sealed with an appropriate amount of DAPI. Image J software quantified the fluorescence intensity.

### Statistics

The result data obtained from this study were analyzed using SPSS software. The t-test was used to compare the experimental results data between two groups, and the one-way/two-way ANOVA or SNK-q test was used to compare the experimental results data between more than two groups. Pearson's correlation analysis was used for correlation analysis, with P < 0.05 as a significant difference. According to the classification of Pearson's correlation coefficient (r), the absolute values of 0–0.30, 0.30–0.50, 0.50–0.70 and 0.70–1.00 indicate "poor" correlation, "fair" or "medium" correlation, "good" correlation and "strong" correlation, respectively. Statistical graphs of all experimental results were produced using Graphpad software and Adobe illustrator software.

## Results

### Fli-1 is involved in the dysfunction of the pulmonary vascular endothelial barrier in ALI

**Figure 1:**
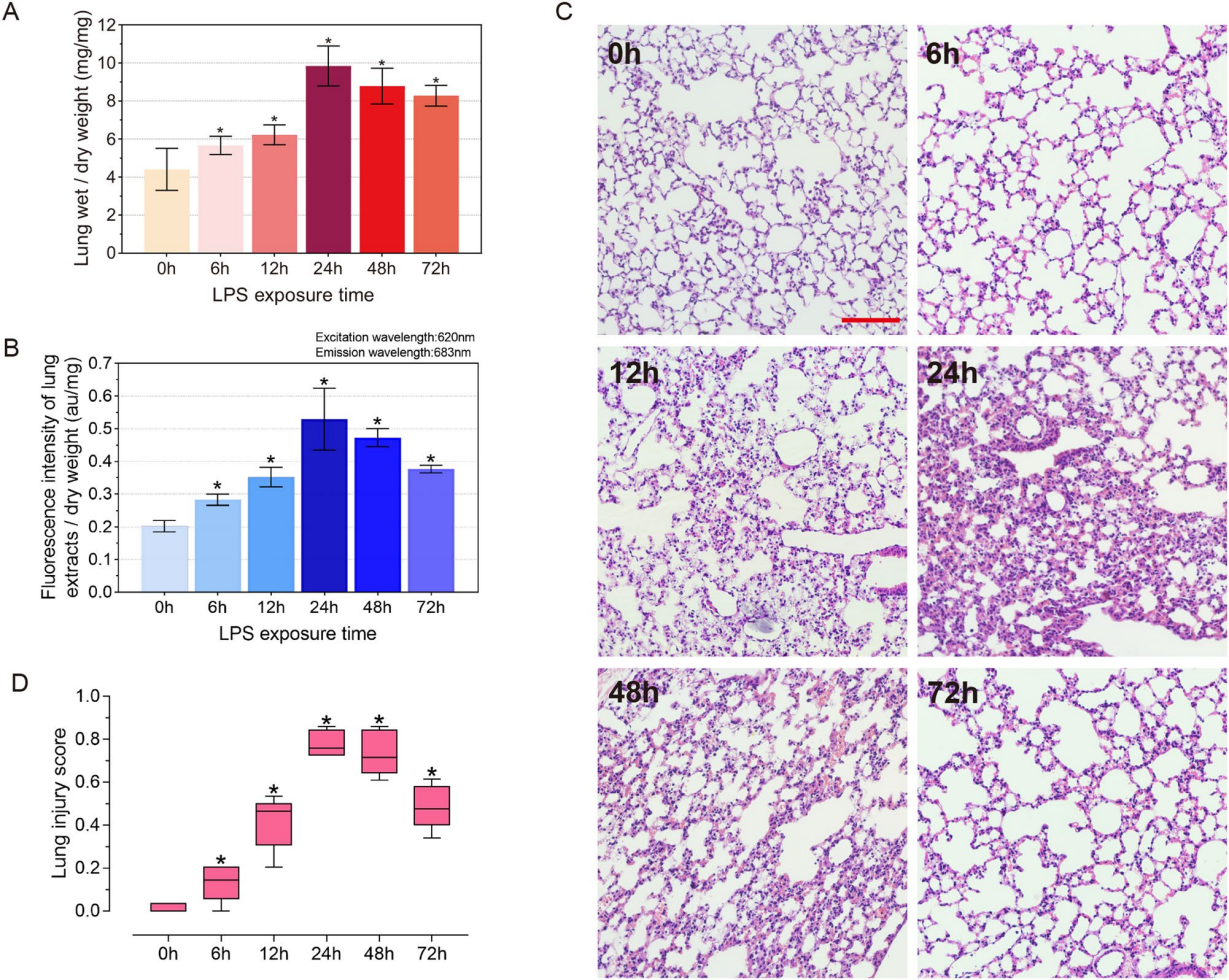
Dynamic time pulmonary function indexes and inflammatory response in ALI mice. (**A**) Wet/dry weight ratio of lungs in mice after LPS treatment at 0, 6, 12, 24, 48 and 72 h (n = 5–10). (**B**) Evans blue assay for lung vascular endothelial permeability in LPS-treated mice at 0, 6, 12, 24, 48 and 72 h (n = 6). (**C**) HE staining of mice lung tissues at 0, 6, 12, 24, 48 and 72 h after LPS treatment (×200 magnification, scale bar: 100 μm for all images). (**D**) Lung injury score at 0–72 h (n = 5). Data are presented as the mean ± SEM. *P < 0.05 vs. the control group.

To investigate the dynamic changes of pulmonary edema in mice after LPS stimulation, we examined the lung wet/dry weight ratio of mice (Fig. 1A). Compared with the 0 h group, the lung wet/dry weight ratio exhibited a significant increase 6 h after LPS injection, and reached its maximum at 24 h. Subsequently, pulmonary edema began to show a recovery trend at 48 h and 72 h. The results of lung vascular endothelial barrier permeability detection by evans blue showed that the lung vascular endothelial barrier permeability increased gradually at a faster rate from 0 to 24 h. In 24–72 h, the endothelial permeability showed a slow recovery phase (Fig. 1B). The morphological changes of lung tissues were stained by HE, and lung injury was quantified by the ATS Lung Injury Scoring System (Fig. 1C,D).

**Figure 2:**
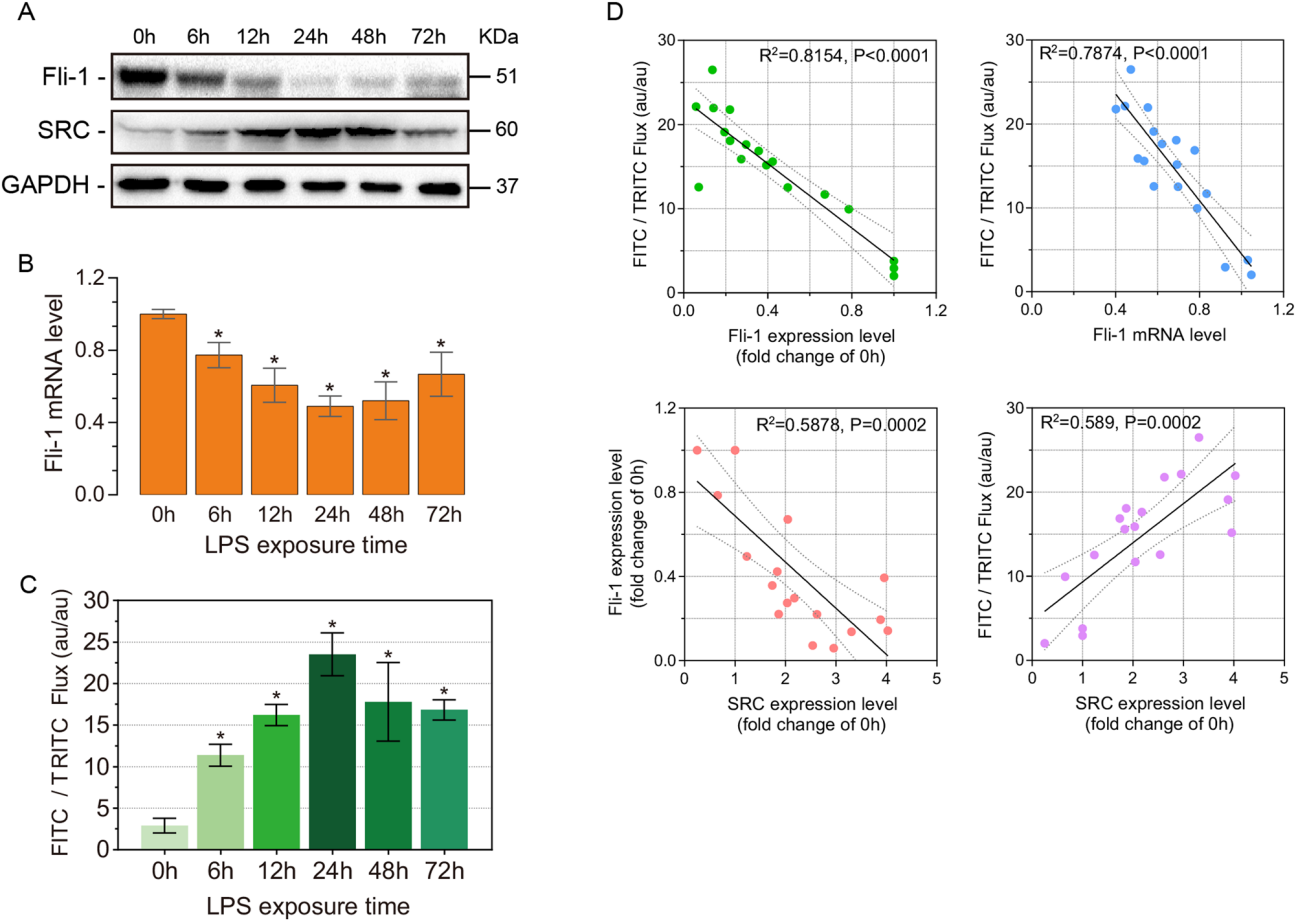
Fli-1 is involved in the process of LPS-induced ALI. (**A**) Fli-1, SRC protein expression levels in lung tissue of mice at 0, 6, 12, 24, 48 and 72 h after LPS treatment (n = 3). Fli-1 original blots are presented in Supplementary Fig. 1. (**B**) Fli-1 mRNA expression level changes (n = 3). (**C**) Dual fluorescent protein tracking assay to detect pulmonary vascular endothelial permeability. (**D**) Pulmonary vascular endothelial permeability was correlated with Fli-1 protein, Fli-1 mRNA and SRC protein, as well as Fli-1 protein—SRC protein. Data are presented as the mean ± SEM. *P < 0.05 vs. the control group.

SRC is a key protein that regulates adherens junctions^13^. Moreover, Fli-1 can inhibit SRC expression^14^. To investigate the relationship between vascular endothelial barrier permeability and Fli-1, we examined the dynamic expression level changes of SRC and Fli-1 after LPS treatment. Western blot showed that Fli-1 protein was continuously down-regulated from 0 to 24 h after LPS treatment, and then started to up-regulated. Meanwhile, SRC protein showed an opposite trend to Fli-1 (Fig. 2A). At the gene level, Fli-1 had the same variation trend as its protein (Fig. 2B). The changes of pulmonary vascular endothelial permeability in mice after LPS treatment were detected using a dual fluorescent protein tracking assay (Fig. 2C). The experimental results validated the conclusions of the evans blue assay. We correlated the dynamic expression changes of Fli-1 protein and SRC protein, and the results of the dual fluorescent protein tracking assay with the dynamic expression levels of Fli-1 protein, Fli-1 gene and SRC protein, respectively, by Pearson correlation analysis. The results showed that all correlation analyses were significant (Fig. 2D).

### Molecular mechanism of apelin against ALI is related to Fli-1

By various dynamic indices, we demonstrated that LPS at a dose of 7.5 mg/kg for 24 h can cause the most severe damage. Therefore, we chose 24 h after LPS treatment as the time point for the detection of each subsequent experiment.

**Figure 3:**
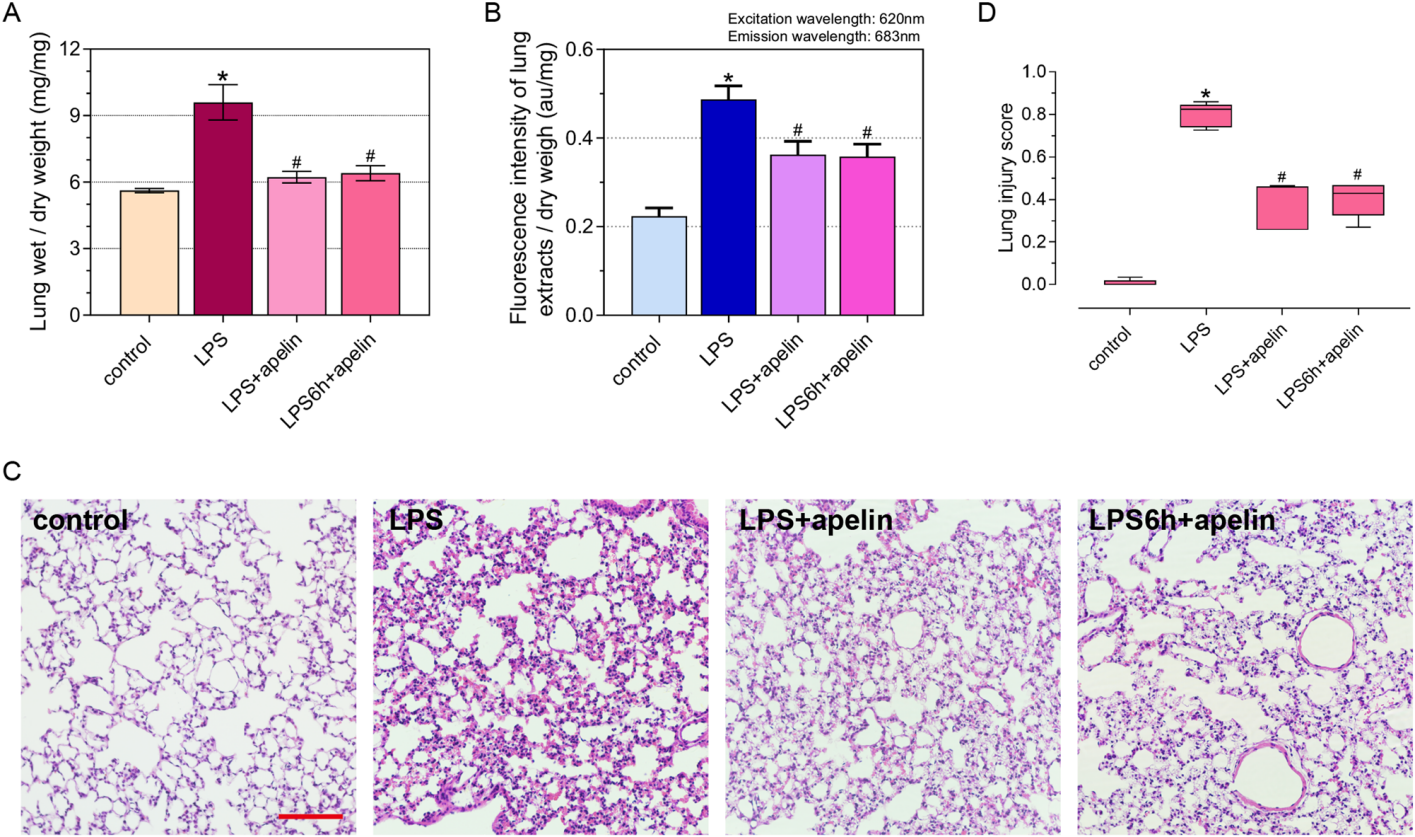
Protective effect of apelin against ALI. (**A**) Lung wet/dry weight ratio to detect changes in whole lung permeability after apelin control effect. (**B**) Evans blue assay to detect changes in pulmonary vascular endothelial permeability after apelin prevention and treatment. (**C**) HE staining demonstrating the inflammatory response of lung tissue after apelin control (×200 magnification, scale bar: 100 μm for all images). (**D**) Lung injury score on HE staining (n = 5). Data are presented as the mean ± SEM. *P < 0.05 vs. the control group, and ^#^P < 0.05 vs. the LPS group.

The lung wet/dry weight ratio was used to evaluate the effect of apelin on whole lung permeability (Fig. 3A). Evans blue measured the pulmonary vascular endothelial permeability in mice after apelin administration (Fig. 3B). Both of these results indicate that the stability of the vascular endothelial barrier was significantly enhanced after apelin prevention and treatment. Histomorphological changes were observed by HE staining (Fig. 3C): after the prevention and treatment with apelin, compared with LPS group, the lung histomorphology of both groups showed obvious relief, edema and exudation were improved, the number of red blood cells decreased, alveoli became loose, and inflammatory cell infiltration in the interstitial tissue decreased. Lung injury scores likewise confirmed (Fig. 3D).

**Figure 4:**
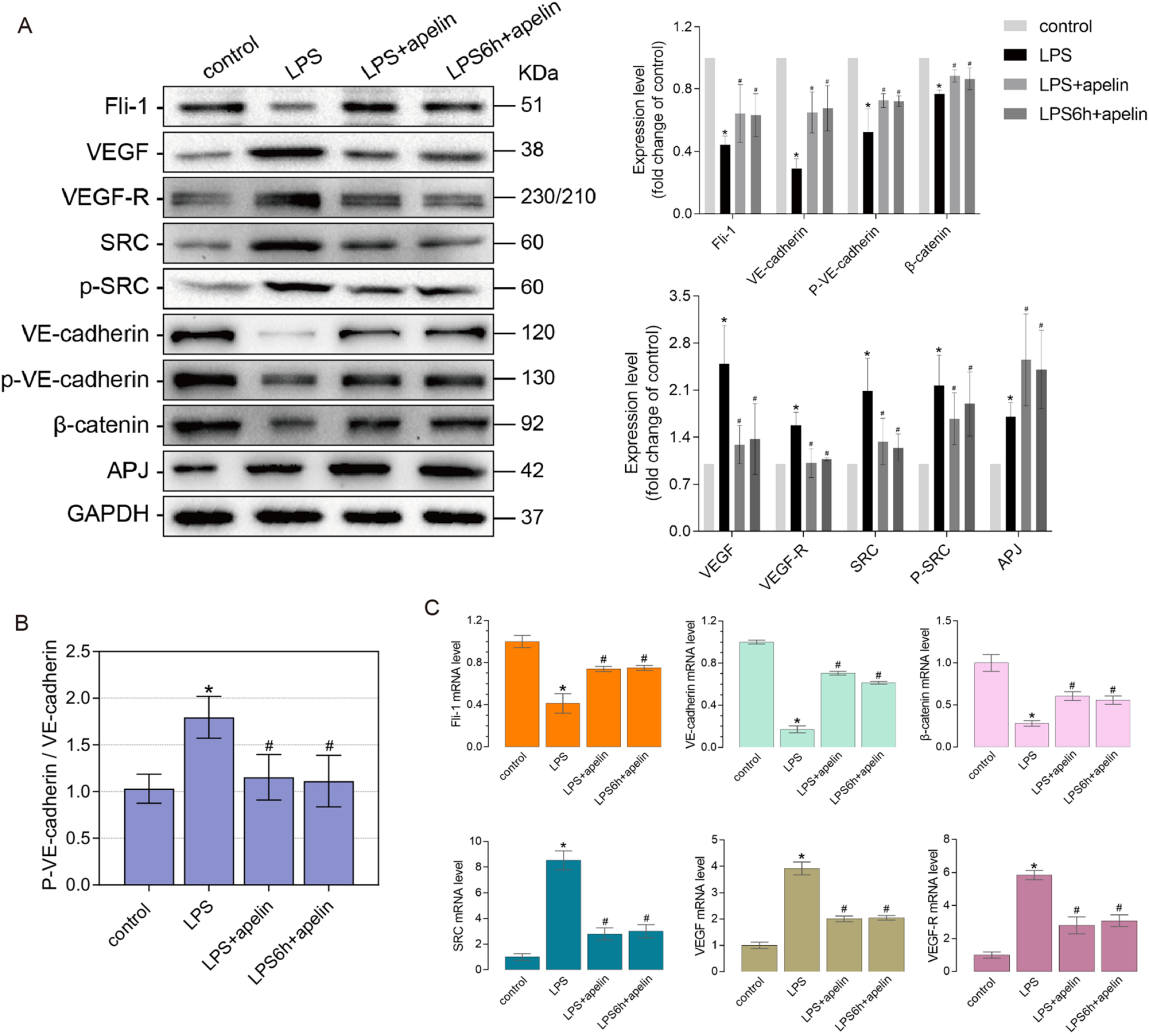
Effect of apelin prevention and treatment on related proteins and genes. (**A**) Changes in Fli-1, APJ and adherens junction-related proteins in lung tissue after prevention and treatment of ALI mice by apelin, detected by western blot (n = 3–4). The bar graphs represent the statistical results of the grayscale values of each group of proteins. (**B**) Effect of apelin prevention and treatment on VE-cadherin internalization (n = 4). (**C**) qRT-PCR detection of apelin on Fli-1 and adherens junction-related protein mRNA levels in lung tissue of ALI mice after prevention and treatment (n = 4). Data are presented as the mean ± SEM. *P < 0.05 vs. the control group, and ^#^P < 0.05 vs. the LPS group.

Western blot results showed in Fig. 4A that the protein expression level of Fli-1 decreased significantly after LPS stimulation. The LPS + apelin group and LPS6h + apelin group showed the same up-regulation trend compared to LPS group. APJ, the receptor of apelin, was up-regulated after LPS stimulation compared with control group. After the use of exogenous apelin for prevention and treatment, the expression of APJ was further increased. Simultaneously, changes in the adherens junction related proteins VE-cadherin, p-VE-cadherin, β-catenin, SRC, p-SRC, VEGF and VEGF-R were detected experimentally. After LPS stimulation, the expression of VE-cadherin, p-VE-cadherin and β-catenin decreased in comparison with control group. And SRC, VEGF and VEGF-R, which caused adherens junction disruption, showed up regulation respectively. In LPS + apelin and LPS6h + apelin groups, VE-cadherin, p-VE-cadherin and β-catenin showed significant up-regulation after apelin treatment compared to LPS group accordingly, and SRC, p-SRC, VEGF and VEGF-R showed significant down-regulation. After LPS stimulation, VE-cadherin was phosphorylated, accompanied by its internalization. However, VE-cadherin and p-VE-cadherin showed the same trend of injury. Therefore, to reflect the degree of VE-cadherin internalization in each group, we used the gray value ratio of p-VE-cadherin to VE-cadherin after western blot development to reflect this (Fig. 4B). The ratio of LPS group was significantly higher than that of control group, indicating that the internalization of VE-cadherin increased after LPS treatment. The ratio of LPS + apelin group was significantly lower than that of LPS + apelin group, indicating that the internalization of VE-cadherin decreased and the stability of the endothelial barrier increased. At the gene level, Fli-1, VE-cadherin, β-catenin, SRC, VEGF and VEGF-R were detected, and the results showed that the trend was consistent with that of protein level (Fig. 4C).

### Apelin against ALI by upregulating Fli-1

**Figure 5:**
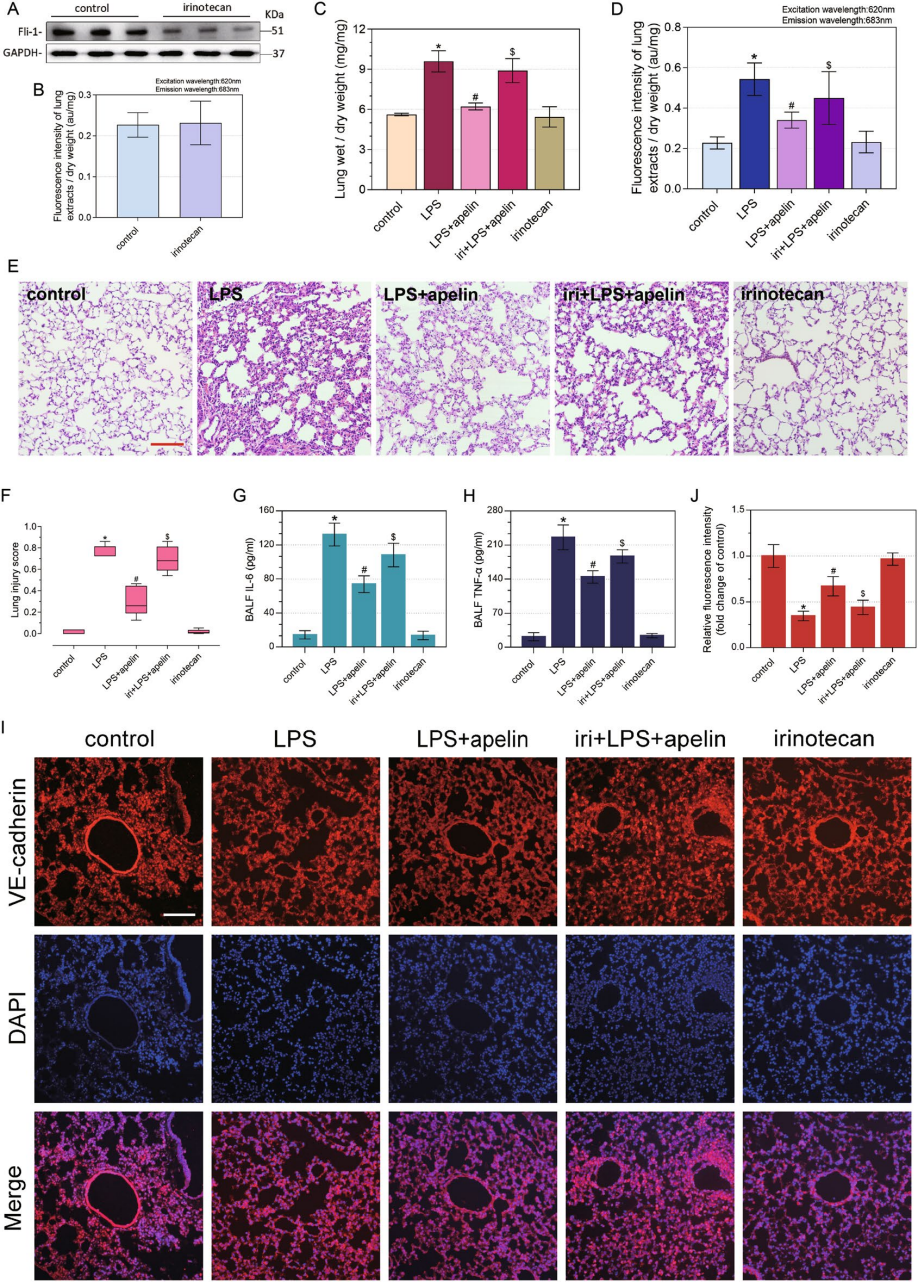
Functional indexes and morphological changes of pulmonary permeability in ALI mice affected by apelin after Fli-1 inhibition. (**A**) Western blot of Fli-1 used to evaluate the inhibitory effect of irinotecan on Fli-1. (**B**) Evans blue assay to evaluate the effect of irinotecan on vascular endothelial permeability. (**C**) Changes in pulmonary vascular endothelial permeability after inhibition of Fli-1, evans blue assay for apelin effect in ALI mice (n = 8). (**D**) Effect of apelin on lung wet/dry weight ratio in ALI mice after inhibition of Fli-1 (n = 4). (**E**) HE staining to observe the inflammatory response of lung tissue treated with apelin in ALI mice, after inhibition of Fli-1 (×200 magnification, scale bar: 100 μm for all images). (**F**) Lung injury score on HE staining (n = 5). (**G**) Levels of inflammatory factor IL-6 in BALF (n = 4). (**H**) Levels of inflammatory factor TNF-α in BALF (n = 4). (**I**) Tissue immunofluorescence staining of VE-cadherin in pulmonary vascular endothelium (×200 magnification, scale bar: 100 μm for all images). Data are presented as the mean ± SEM. *P < 0.05 vs. the control group, ^#^P < 0.05 vs. the LPS group and ^$^P < 0.05 vs. the LPS + apelin group. (**J**) Relative fluorescence intensity of VE-cadherin in pulmonary vascular endothelium (n = 3).

Irinotecan was selected as an effective method to regulate the decrease of Fli-1. To start with, we evaluated the inhibitory effect of irinotecan. Mice were treated with irinotecan for 7 days. Western blot showed that irinotecan could effectively inhibit the expression of Fli-1 (Fig. 5A). To exclude the effect of inhibitors on pulmonary vascular endothelial permeability, the pulmonary vascular endothelial permeability of irinotecan group and control group was examined using evans blue (Fig. 5B). There was no statistical difference in pulmonary vascular endothelial permeability in irinotecan group compared with the control group (P > 0.05), indicating that irinotecan had no effect on vascular endothelial permeability.

To investigate the inflammatory exudative status of the lungs after Fli-1 inhibition, we examined the functional indices and morphological characteristics of the lungs. The results of the lung wet/dry weight ratio showed no significant difference in irinotecan group compared to control group (P > 0.05), while the iri + LPS + apelin group showed an increase in wet/dry weight ratio compared to LPS + apelin group (P < 0.05), and the whole lung permeability was significantly increased (Fig. 5C). In addition, the lung vascular endothelial permeability was measured by evans blue (Fig. 5D). Compared with LPS + apelin group, the permeability of pulmonary vascular endothelial cells of iri + LPS + apelin group increased. Moreover, there was no statistical difference in pulmonary vascular endothelial permeability between iri + LPS + apelin group and LPS group (P > 0.05). HE staining and the lung injury score also demonstrated that the inflammatory response tended to be severe after the suppression of Fli-1 (Fig. 5E,F). In contrast to LPS + apelin group, the lung tissue inflammatory response was significantly enhanced in iri + LPS + apelin group, as evidenced by increased tissue interstitial edema, exudation and inter alveolar adhesions, with erythrocytes scattered in the alveoli and tissue interstitial. A large number of inflammatory cells infiltrated, and the inflammatory response was significantly more serious than that of LPS + apelin group. The inflammatory factors IL-6 and TNF-α in BALF were significantly up-regulated after LPS stimulation, and down-regulated after the anti-inflammatory and protective effect of apelin. However, the effect of apelin on inflammatory factor levels was greatly attenuated after inhibition of Fli-1 (Fig. 5G,H). VE-cadherin tissue immunofluorescence staining and its relative fluorescence intensity reflect the strength of pulmonary vascular endothelial adherens junctions (Fig. 5I,J).

**Figure 6:**
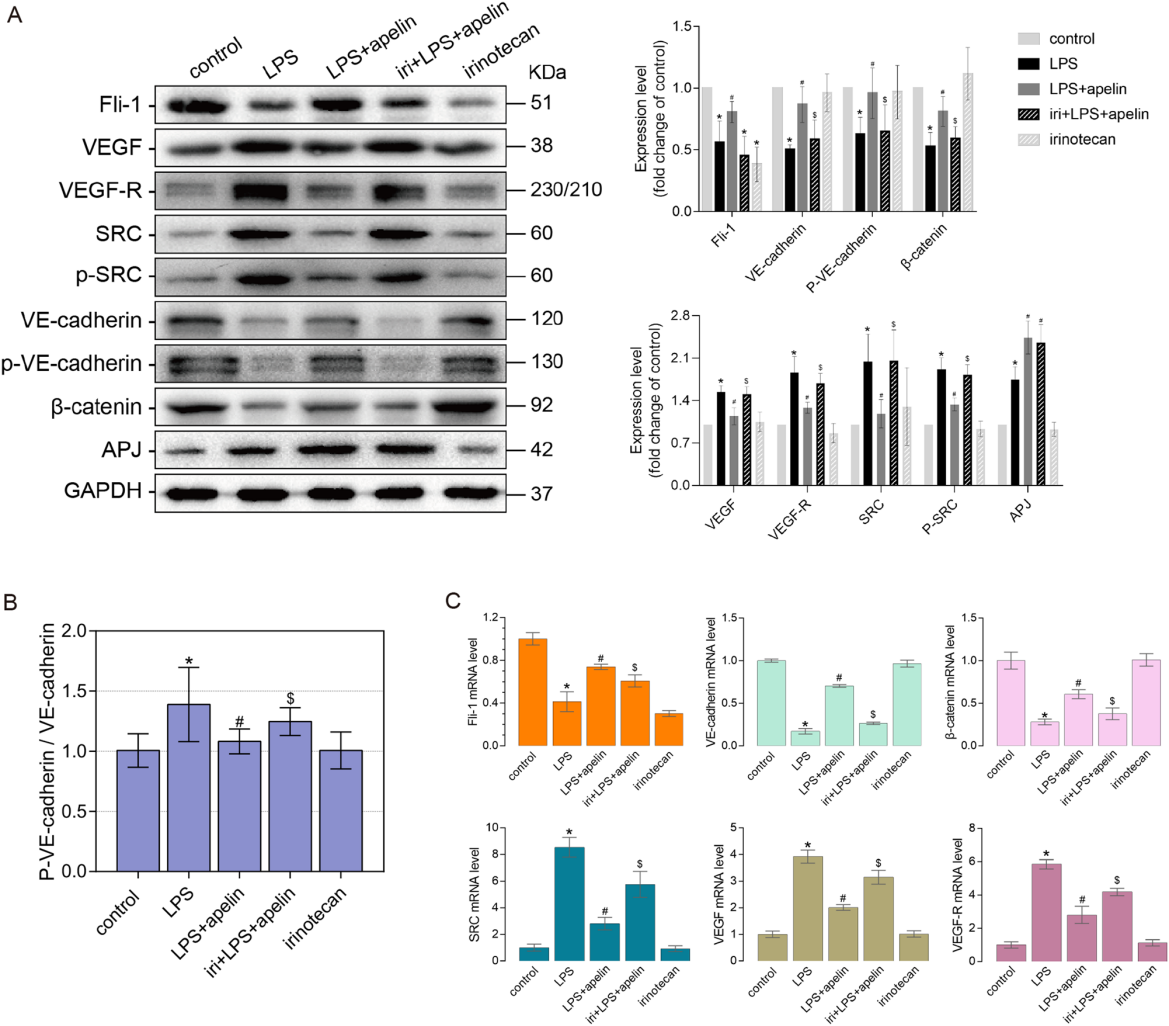
The effects of apelin on pulmonary vascular endothelial adherens junction-related proteins and genes in ALI mice after inhibition of Fli-1. (**A**) Effect of apelin on lung tissue Fli-1, APJ and adherens junction-related proteins in ALI mice by western blot after inhibition of Fli-1. (**B**) Inhibition of Fli-1, the degree of internalization of VE-cadherin in each group (n = 3). (**C**) qRT-PCR to detect the effect of apelin on mRNA levels of Fli-1 and adherens junction-related proteins in lung tissue of ALI mice after Fli-1 inhibition (n = 4). Data are presented as the mean ± SEM. *P < 0.05 vs. the control group, ^#^P < 0.05 vs. the LPS group and ^$^P < 0.05 vs. the LPS + apelin group.

Compared with LPS group, the expression levels of APJ in LPS + apelin and iri + LPS + apelin groups was further up-regulated. In the irinotecan group, the expression levels of adherens junction related proteins were similar to those in control group (P > 0.05). Comparing the iri + LPS + apelin group with the LPS + apelin group, it was found that VE-cadherin, p-VE-cadherin and β-catenin were significantly down-regulated and SRC, p-SRC, VEGF and VEGF-R showed elevated expression after Fli-1 inhibition (Fig. 6A). Comparison of p-VE-cadherin with VE-cadherin grayscale values showed that the internalization of VE-cadherin increased after inhibitor treatment compared with LPS + apelin group (Fig. 6B). The gene expression levels of Fli-1 and adherens junction related proteins were detected, and the results were consistent with the trend of proteins (Fig. 6C).

## Discussion

Our study shows that dysfunction of the pulmonary vascular endothelial barrier is a vital link in the induction of ALI by LPS. During the course of ALI, the pulmonary vascular endothelial barrier was quickly destroyed, reaching the most serious level at 24 h. Subsequently, due to the initiation of endogenous repair mechanisms, ALI started to enter the recovery period, and the vascular endothelial barrier gradually improved. Moreover, the expression level of Fli-1 was significantly correlated with lung wet/dry weight ratio, vascular endothelial permeability, and adherens junction related protein expression. This suggests that Fli-1 is involved in the process of vascular endothelial barrier dysfunction in ALI. We also found that apelin has a desirable preventive and therapeutic effect on ALI. It can regulate the expression of adherens junction related proteins, reduce the phosphorylation and internalization of VE-cadherin, and enhance the stability of adherens junctions, thereby repairing the functional state of the vascular endothelial barrier, improving whole lung permeability, and reducing the inflammatory reaction. Surprisingly, apelin was found to be accompanied by the up regulation of Fli-1 in the prevention and treatment of ALI. This suggests that Fli-1 may be involved in the restoration of apelin. By inhibiting Fli-1 with irinotecan, the protective function of apelin was diminished, vascular endothelial barrier function was not improved, and the expression level of adherens junction proteins was not corrected by apelin. These results demonstrate that the molecular mechanism of apelin's anti-ALI is through the up regulation of Fli-1, which regulates the expression of adherens junction proteins and eventually repairs the function of vascular endothelial barrier.

The inter endothelial cell junctions, the tight adhesion between endothelial cells and basement membrane, and the shape of endothelial cells play important roles in maintaining the stability and integrity of the endothelial barrier^15^. The adherens junctions consist of VE-cadherin and associated α-catenin, β-catenin and p120-catenin adhesion complexes^16^. VE-cadherin anchors to circumferential actin bundles (CABs) via α-catenin and β-catenin linker proteins thus making cell junctions to remain stable between them. When the permeability-increasing drugs are used, the formation of cytoplasmic stress fibers and the contraction of endothelial cell will be induced. The VE-cadherin, which constitutes the adherens junctions, is subjected to increased tension, local junctions are destroyed, and vascular permeability is increased^3,4^. LING-YAN LIU et al. found that the endothelial permeability of ALI induced by LPS showed dynamic changes by double fluorescent protein tracking assay, and reached the maximum endothelial permeability at 24 h^17^. In our research, we got the same conclusion through the evans blue assay. SRC plays an important role in maintaining homeostasis of the pulmonary vascular endothelial barrier. It can be activated in multiple ways to become p-SRC, which can activate specific signaling pathways through phosphorylation of target proteins^18^. P-SRC phosphorylates myosin light chain or disrupts the junctions of VE-cadherin and associated proteins such as p120-catenin and β-catenin^19^. In this experiments, SRC was positively correlated with vascular endothelial permeability from 0 to 72 h. Fli-1 is a member of the ETS transcription factor family, which mainly exists in vascular endothelial cells, macrophages, immune cells, hematopoietic system and fibroblasts^20^. It participates in the regulation of inflammatory reaction, embryo development, cell proliferation, differentiation and migration^21^. Our results showed that the expression level of Fli-1 in mouse lungs after LPS stimulation was significantly correlated with vascular endothelial permeability and SCR. This suggests that Fli-1 is involved in the dysregulation of vascular endothelial barrier function in ALI and even in regulating the expression of adherens junction related proteins.

Apelin is a small molecular peptide mainly expressed in endothelial cells. APJ is the endogenous receptor of apelin, and apelin-13 is the most active ligand of APJ^22^. In LPS-induced ALI, apelin can effectively improve the inflammatory response of lung tissue and reduce mortality in mice^23^. However, the exact molecular mechanism by which apelin acts is not yet clear. To investigate the relationship between Fli-1 and the apelin/APJ axis, after ALI was controlled by apelin, the expression level of Fli-1 was detected. The results showed that the inflammatory response of lung tissue was alleviated after apelin prevention and treatment. The Fli-1 protein level was significantly increased compared to LPS group and accompanied by up-regulation of VE-cadherin, p-VE-cadherin, β-catenin as well as down-regulation of SRC, p-SRC, VEGF and VEGF-R. SRC and p-SRC showed the same trend indicating that in ALI the activity of SRC changes in parallel with the protein level. APJ was significantly increased after LPS due to the activation of endogenous repair mechanism. The LPS + apelin group and LPS6h + apelin group showed further elevation of APJ after exogenous apelin agonism. It indicates that apelin exerts its protective effect against ALI by regulating adherens junction-related proteins for promoting vascular endothelial barrier repair. The VE-cadherin tissue immunofluorescence results reflect this role of apelin more visually from a morphological perspective. Moreover, apelin affected the expression of Fli-1 during this process, suggesting that Fli-1 is involved in the repair mechanism of apelin. In addition, we found no significant difference between LPS + apelin and LPS6h + apelin groups in all experimental results. This suggests that apelin has a desirable preventive and therapeutic effect on ALI. The possible reason is that the inflammation caused by LPS 6 h is in its early stages, and apelin has a similar effect on the prevention of ALI and early inflammation treatment.

To demonstrate the molecular mechanism of apelin's preventive and therapeutic effects, Fli-1 inhibitor was used to conduct a reverse validation experiment. The topoisomerase I inhibitor camptothecin (CPT) can effectively inhibit the transcription activity of Fli-1 at very low concentration. There have been animal experiments demonstrating a strong tolerance to it in mice^24^. Irinotecan (CPT-11) is a semi synthetic derivative of CPT^25^. Our experiments confirmed that irinotecan can stably and effectively inhibit Fli-1 expression in mouse lung tissues both at gene and protein levels. Moreover, irinotecan irinotecan had no obvious effects on the pulmonary vascular endothelial barrier by the evans blue assay. Further, under the condition of low expression of Fli-1, the results of various assays showed that the protective effect of apelin was substantially diminished. This indicates that it is difficult for apelin to fully mobilizing the repair mechanisms of the vascular endothelial barrier after the inhibition of Fli-1. More importantly, these results demonstrate that Fli-1 is a pivotal link in the molecular mechanism of apelin against ALI. LPS-induced ALI is mainly mediated through the NF-κB pathway and produces a range of inflammatory factors^26^. The results of inflammatory factor assay in BALF showed that up regulated Fli-1 could effectively reduce the production of inflammatory factors and thus exert a protective effect against LPS. Vascular endothelial growth factor (VEGF), also known as a vascular permeability factor, is another important protein for regulating vascular endothelium. It not only induces angiogenesis but also mediates the disruption of the vascular barrier, resulting in vascular leakage^27^. VEGF receptor-2 (VEGFR-2) phosphorylates tyrosine at the Y658 and Y731 sites of VE-cadherin by inducing SRC family kinases, leading to destabilization of adherens junctions and increased endothelial cell permeability^28,29^. Youfen Ma et al. found that the up-regulation of Fli-1 significantly inhibited VEGF expression^6^. In this experiment, both the LPS group and iri + LPS + apelin group showed similar results with low levels of Fli-1 with down-regulation of VE-cadherin, p-VE-cadherin, β-catenin and up-regulation of SRC, p-SRC, VEGF and VEGF-R. Therefore, we conclude that the molecular mechanism of apelin repair of the vascular endothelial barrier in ALI is achieved by up-regulating Fli-1. Fli-1 down regulates the expression of VEGF, VEGF-R, SRC and p-SRC, resulting in increased expression of proteins that constitute adherens junctions, reduced VE-cadherin internalization and enhanced endothelial barrier stability.

Previous studies have found that the apelin-APJ pathway is an endogenous anti-injury mechanism with protective effects against ALI^9^. However, this study did not further explore the molecular mechanism of apelin's anti-ALI. Our research first discusses the intrinsic molecular mechanism of apelin's role in protecting adherens junctions from the perspective of the vascular endothelial barrier. Due to the unpredictability of ALI, inflammation often occurs earlier than detection and treatment. Therefore, this study explored the experimental results of preventive and early treatment of inflammation. By dynamic time detection, we identified 24 h as the most severe time point for LPS-induced endothelial barrier function destruction in ALI mice. In parallel, we identified the involvement of Fli-1 in the occurrence of ALI. More surprisingly, in the process of apelin prevention and treatment of ALI, we found that Fli-1 is not only involved in the damage but also may be related to the molecular mechanism of repair. Subsequently, the study used irinotecan, an inhibitor of Fli-1, to reverse the molecular mechanism underlying the regulation of Fli-1 by apelin to improve the stability of adherens junctions.

In the present study, we only used Fli-1 inhibitors to validate the mechanism, but did not overexpress Fli-1 to assist in demonstrating the relationship between apelin and Fli-1. This is because the latest published literature has not reported that an effective Fli-1 agonist does not affect the stability of adherens junctions. Gene knockout mice can eliminate the interference of the target genes more completely than inhibitors, and can avoid the other side effects of inhibitors, thus ensuring the stability of the experimental model. However, at present, it is not yet possible to generate knockout mice that specifically knock out Fli-1 in the lung organs of mice. Therefore, further validation experiments using knockout mice are also not available for this experiment.

## Conclusion

In a word, this study revealed the role of the small molecule peptide apelin in preventing LPS-induced ALI vascular endothelial barrier dysfunction and its molecular mechanism. The study showed that Fli-1 was involved in the dysfunction of the vascular endothelial barrier in ALI through a series of animal experiments. More importantly, apelin plays a role in the prevention and treatment of pulmonary vascular endothelial barrier dysfunction by upregulating Fli-1. This study illustrates apelin in regulating regulates vascular endothelial adherens junctions and the key role played by Fli-1 from the in vivo functional level to the molecular molecular level. These findings provide new way to reduce the incidence of clinical acute lung injury and improve its survival rate, and lay the foundation for subsequent study of therapeutic targets and drug development for acute lung inflammation.

## Supplementary Information

Supplementary Information 1.

Supplementary Information 2.

## Data Availability

The datasets generated during and analysed during the current study are available from the corresponding author on reasonable request.
