# Supplementary material for: Effect and mechanism of apelin on lipopolysaccharide induced acute pulmonary vascular endothelial barrier dysfunction
Source: Sci Rep. 2023 Jan 27;13:1560. doi: 10.1038/s41598-023-27889-6 (PMC9883263; doi:10.1038/s41598-023-27889-6)

control group VE-cadherin

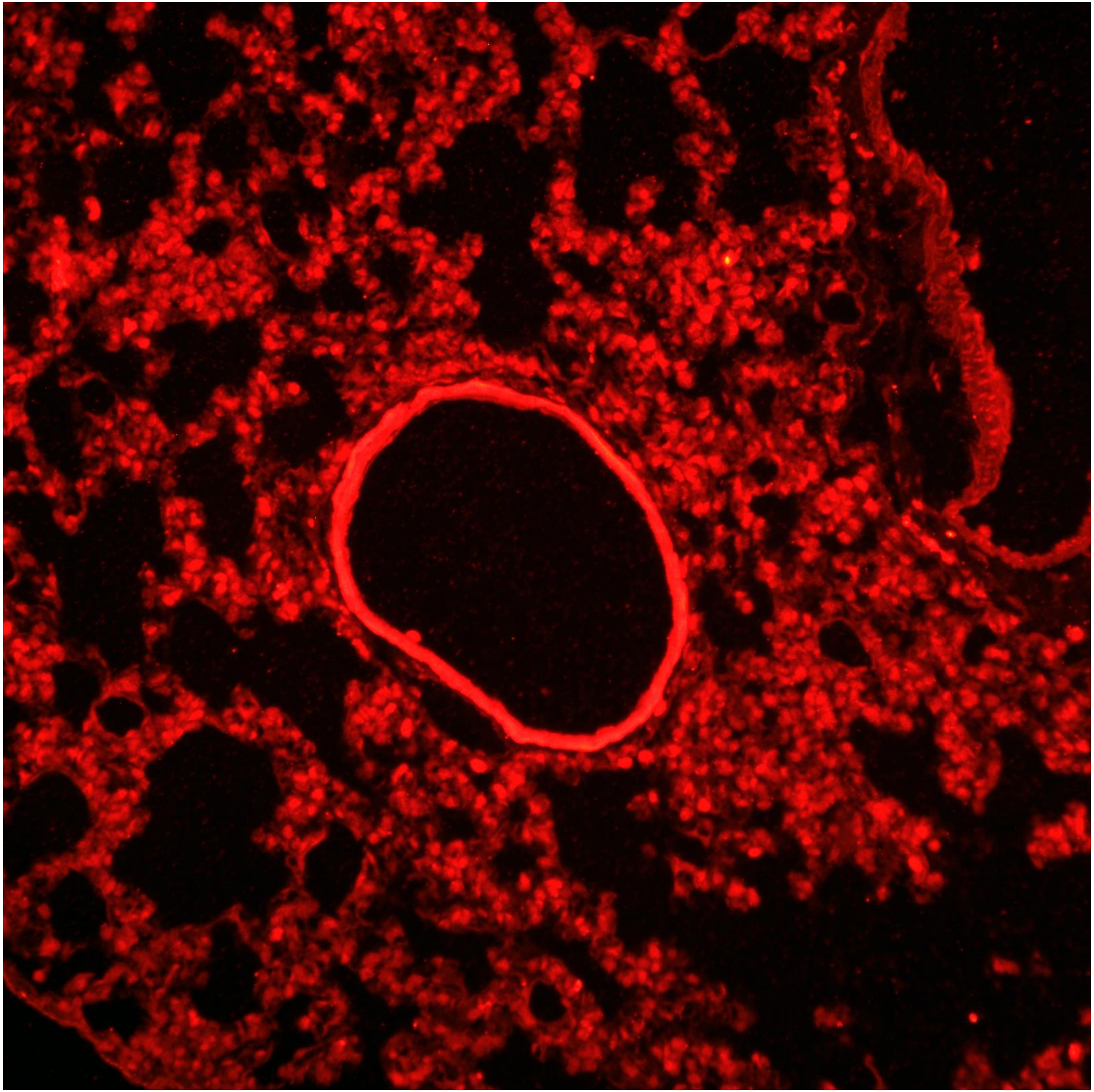

control group DAPI

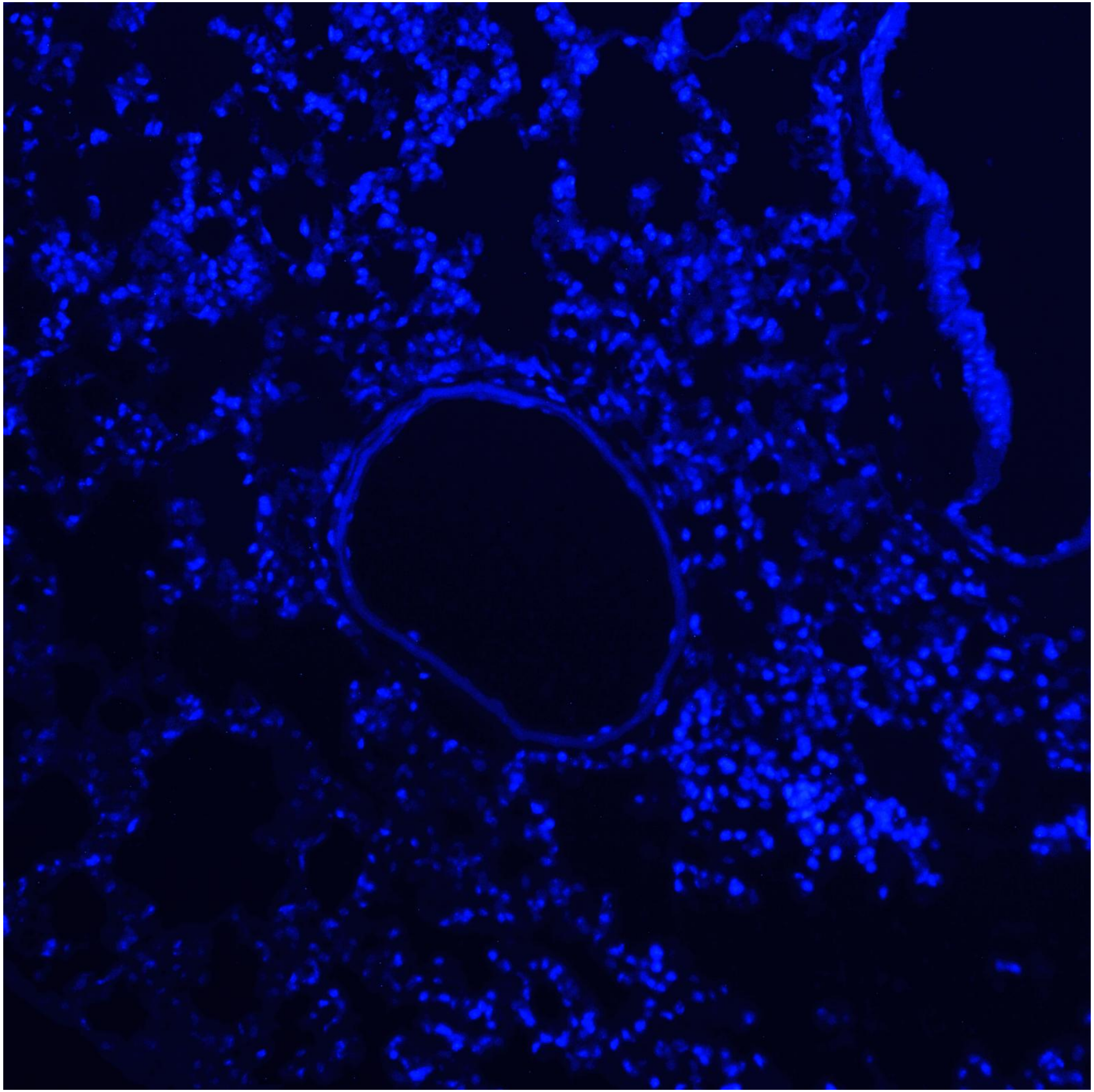

control group Merge

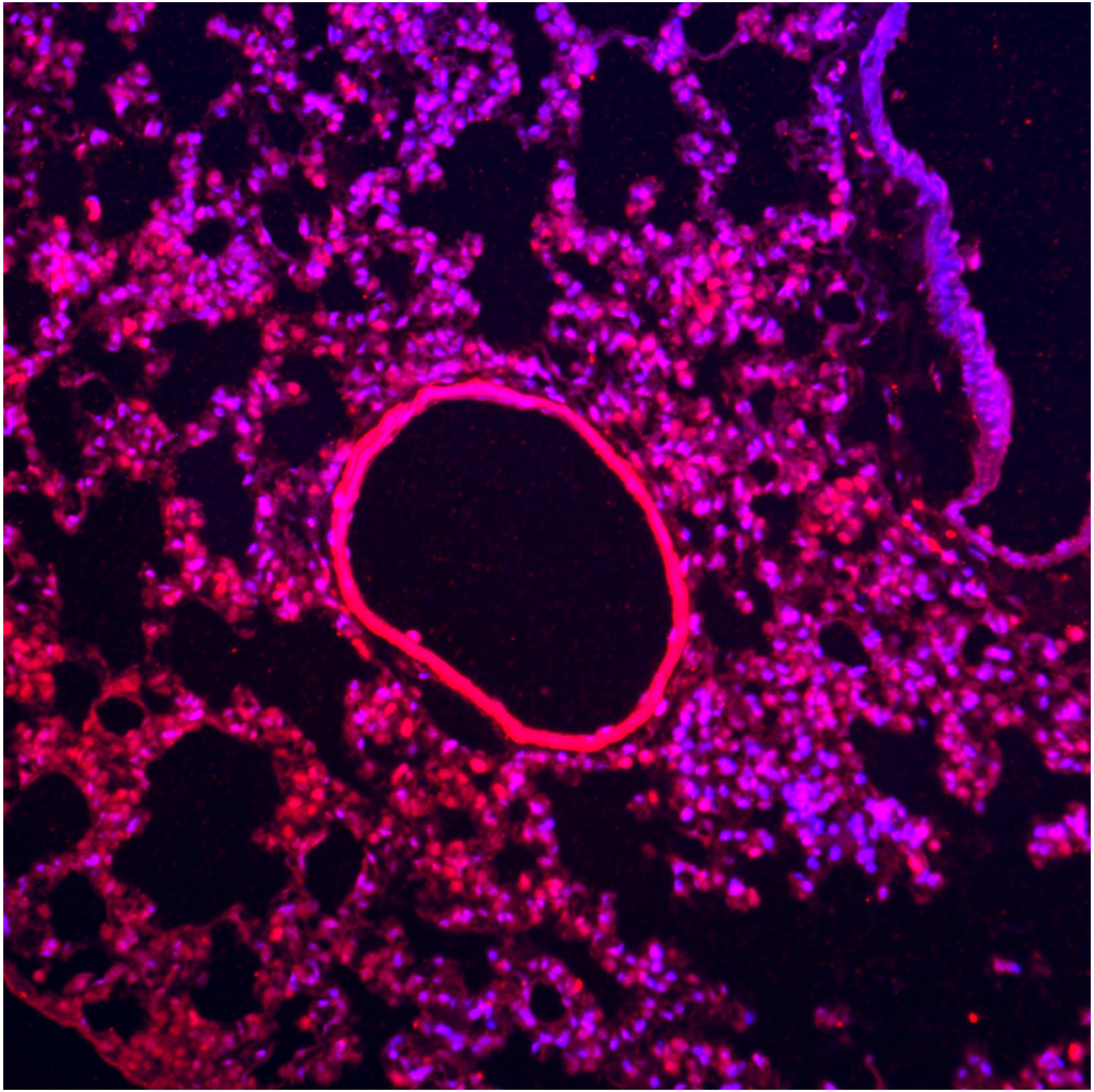

LPS group VE-cadherin

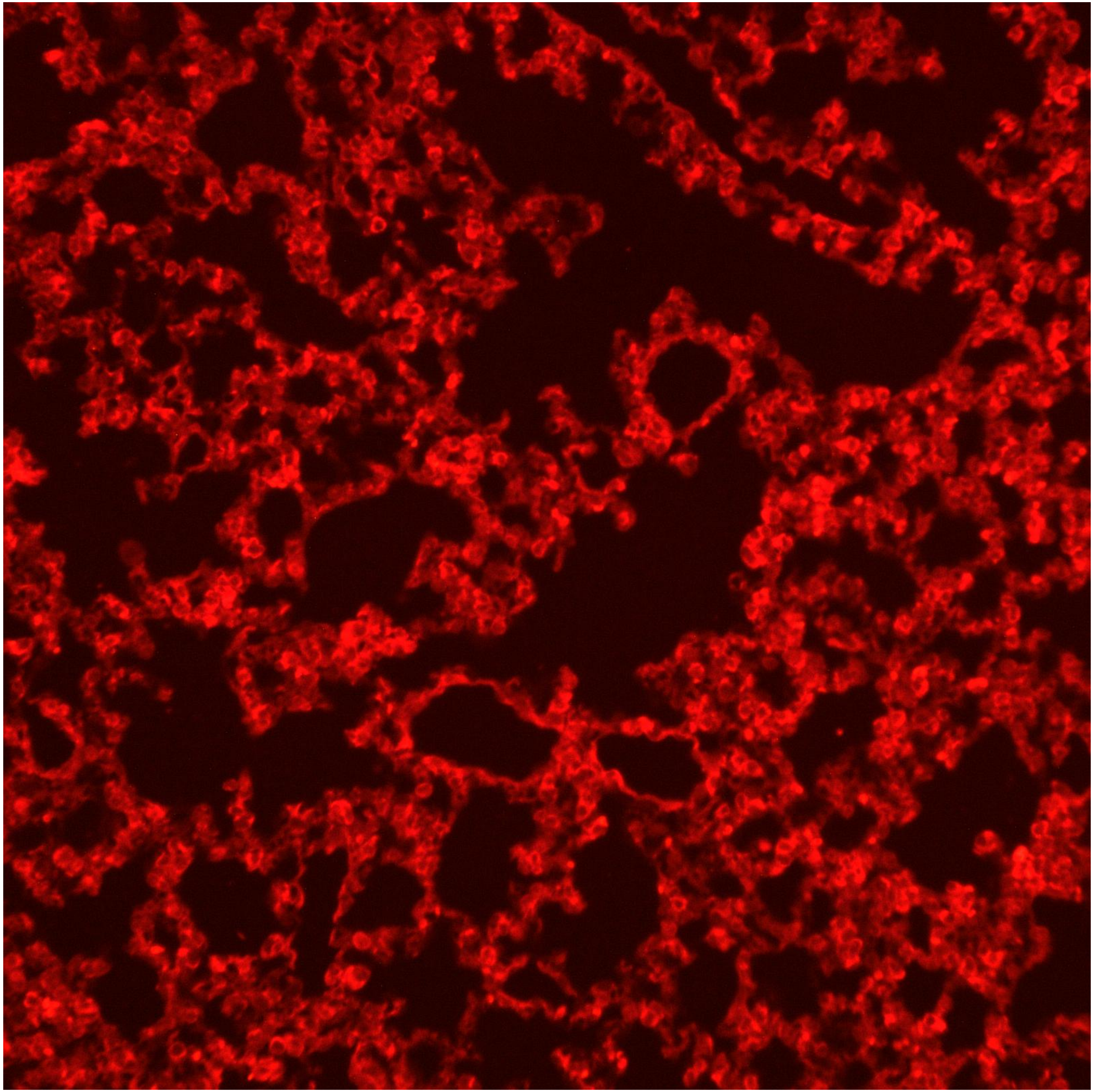

LPS group DAPI

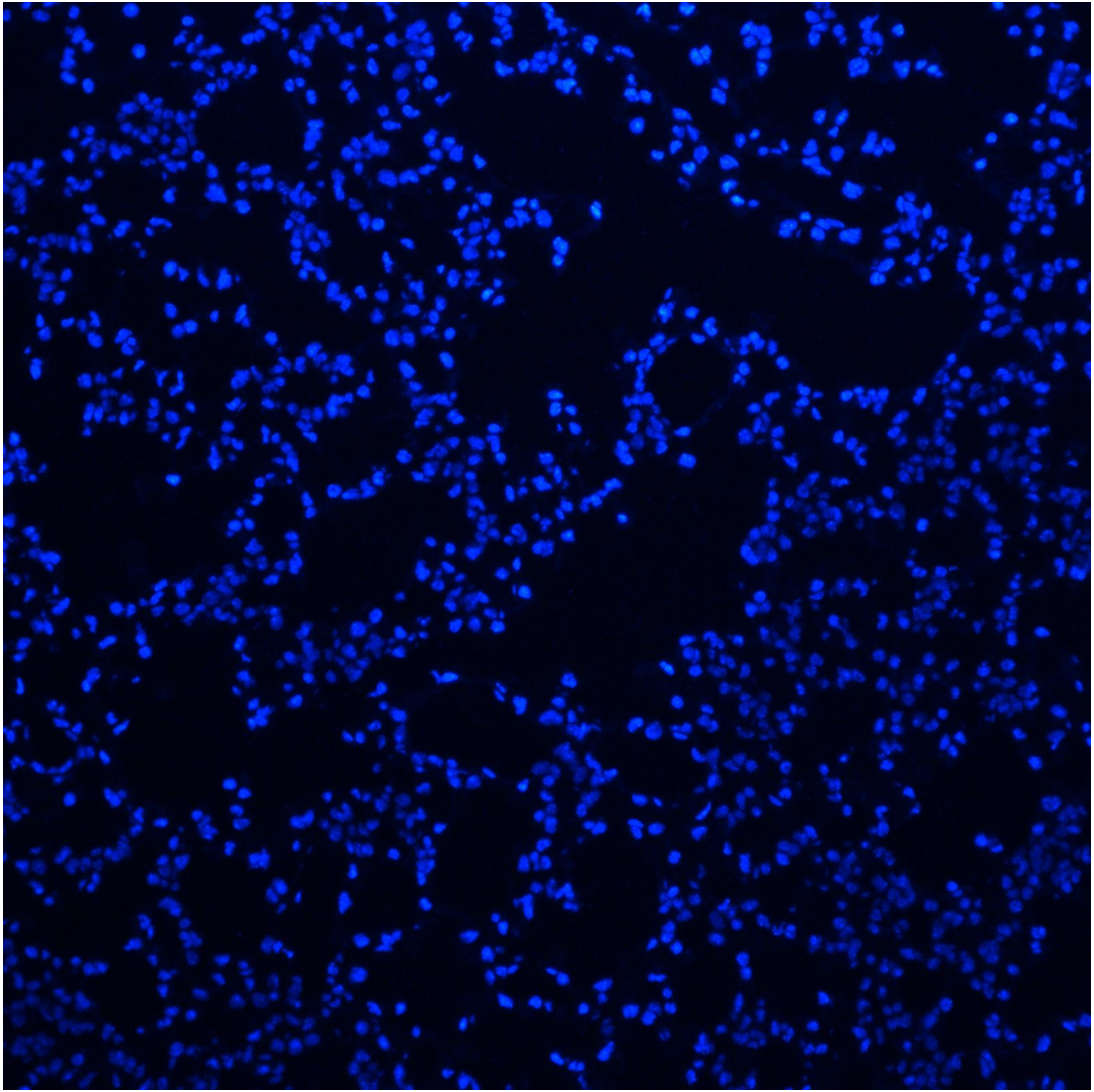

LPS group Merge

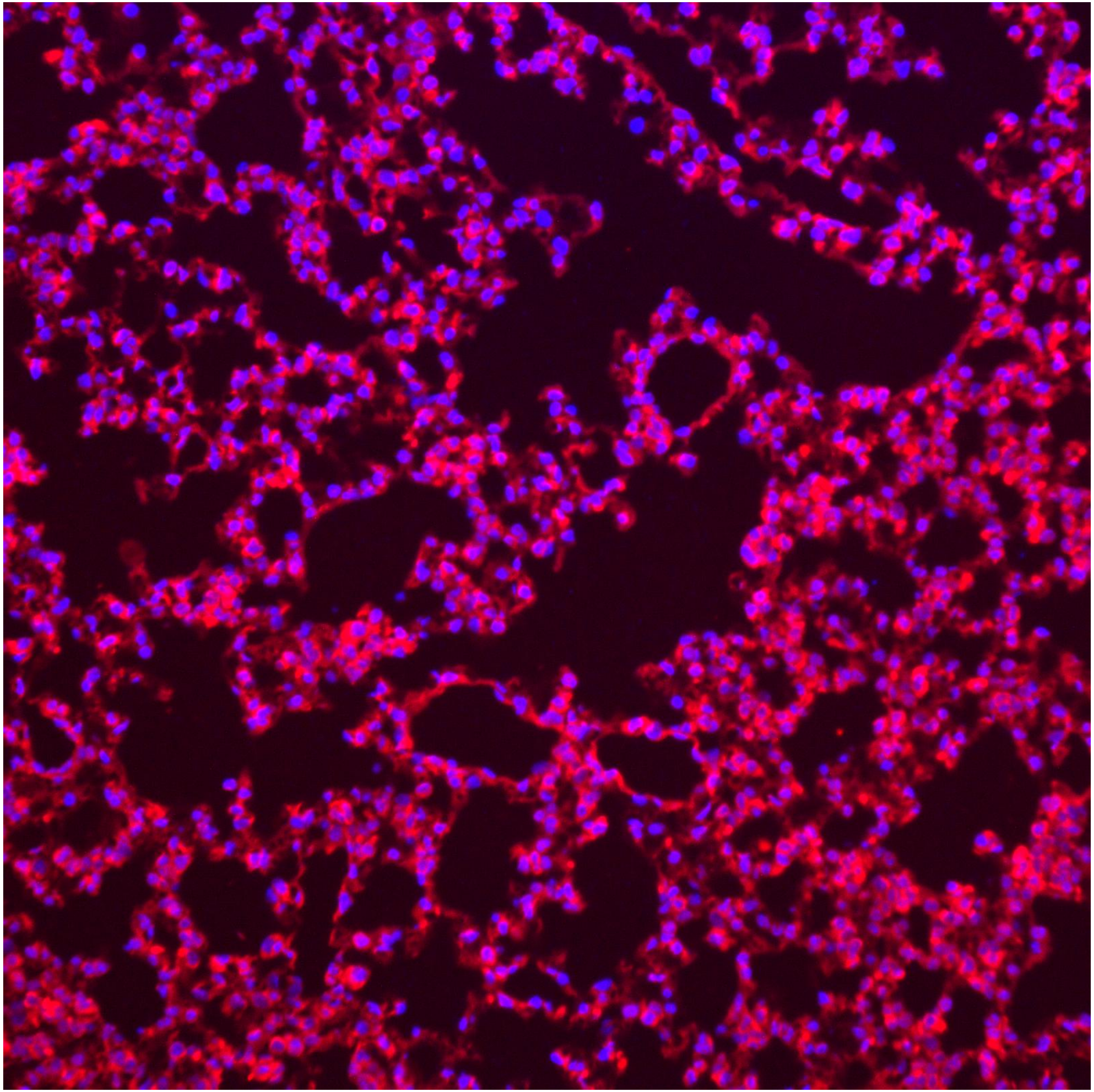

LPS+apelin group VE-cadherin

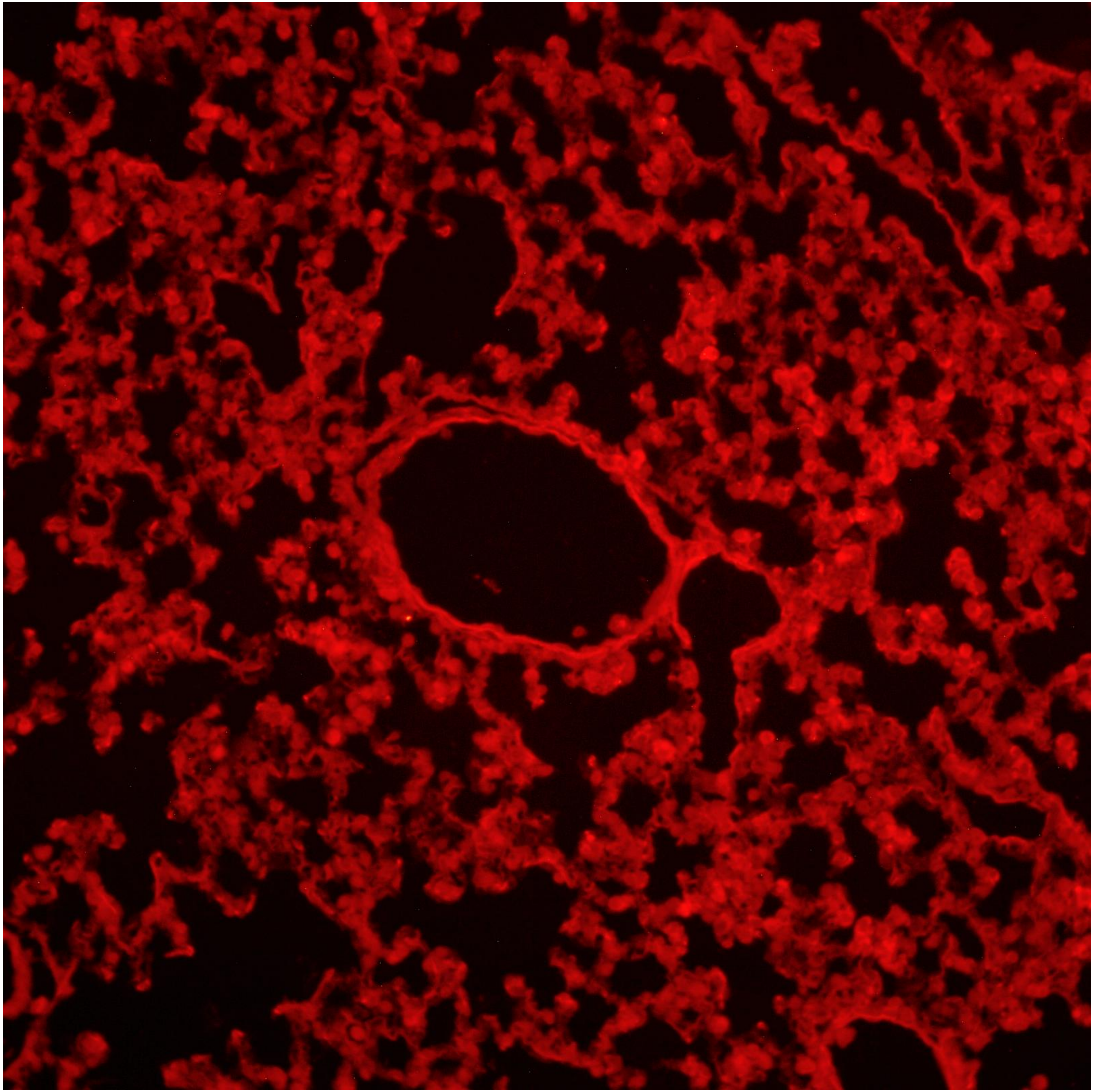

LPS+apelin group DAPI

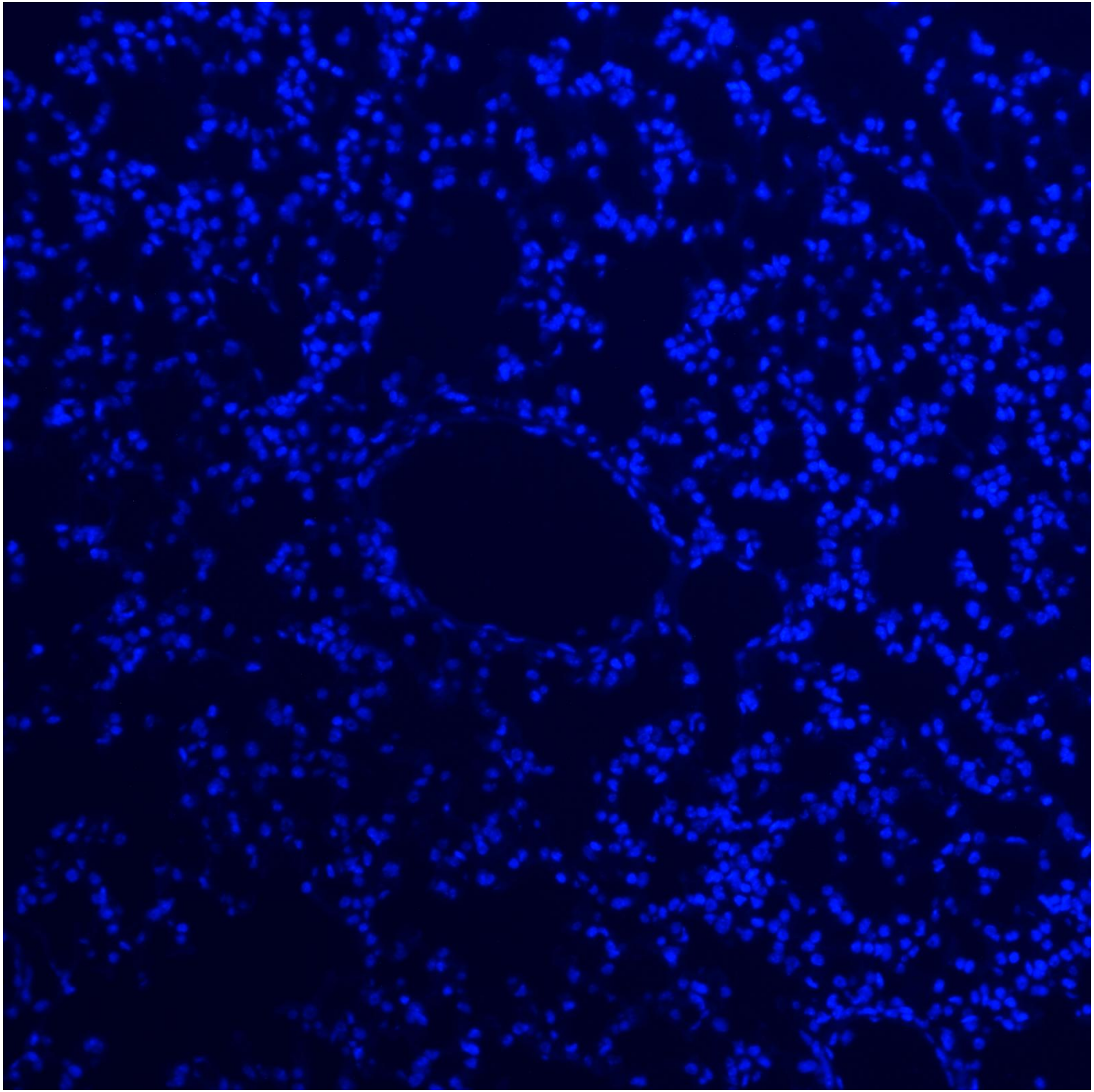

LPS+apelin group Merge

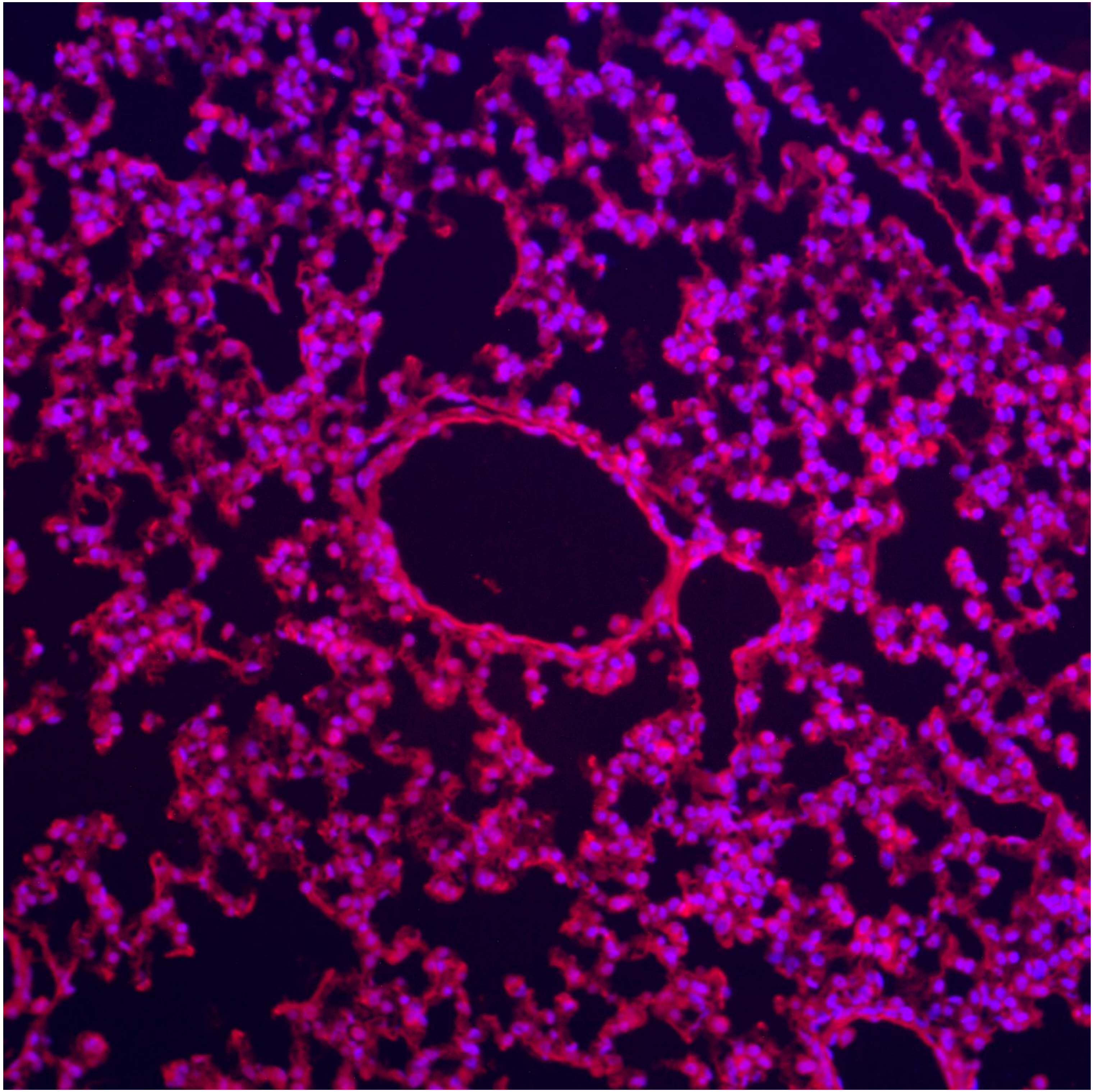

iri+LPS+apelin group VE-cadherin

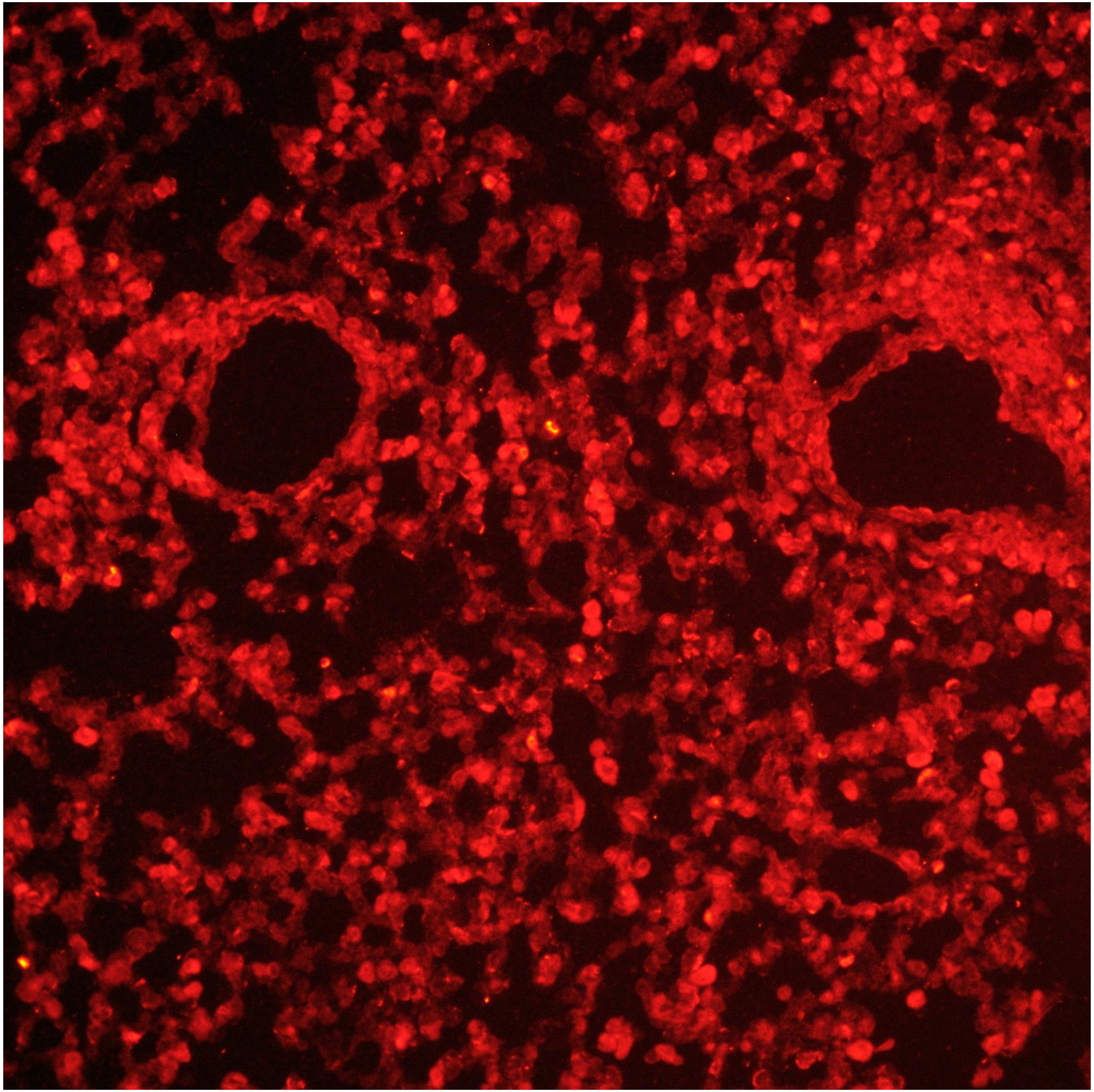

iri+LPS+apelin group DAPI

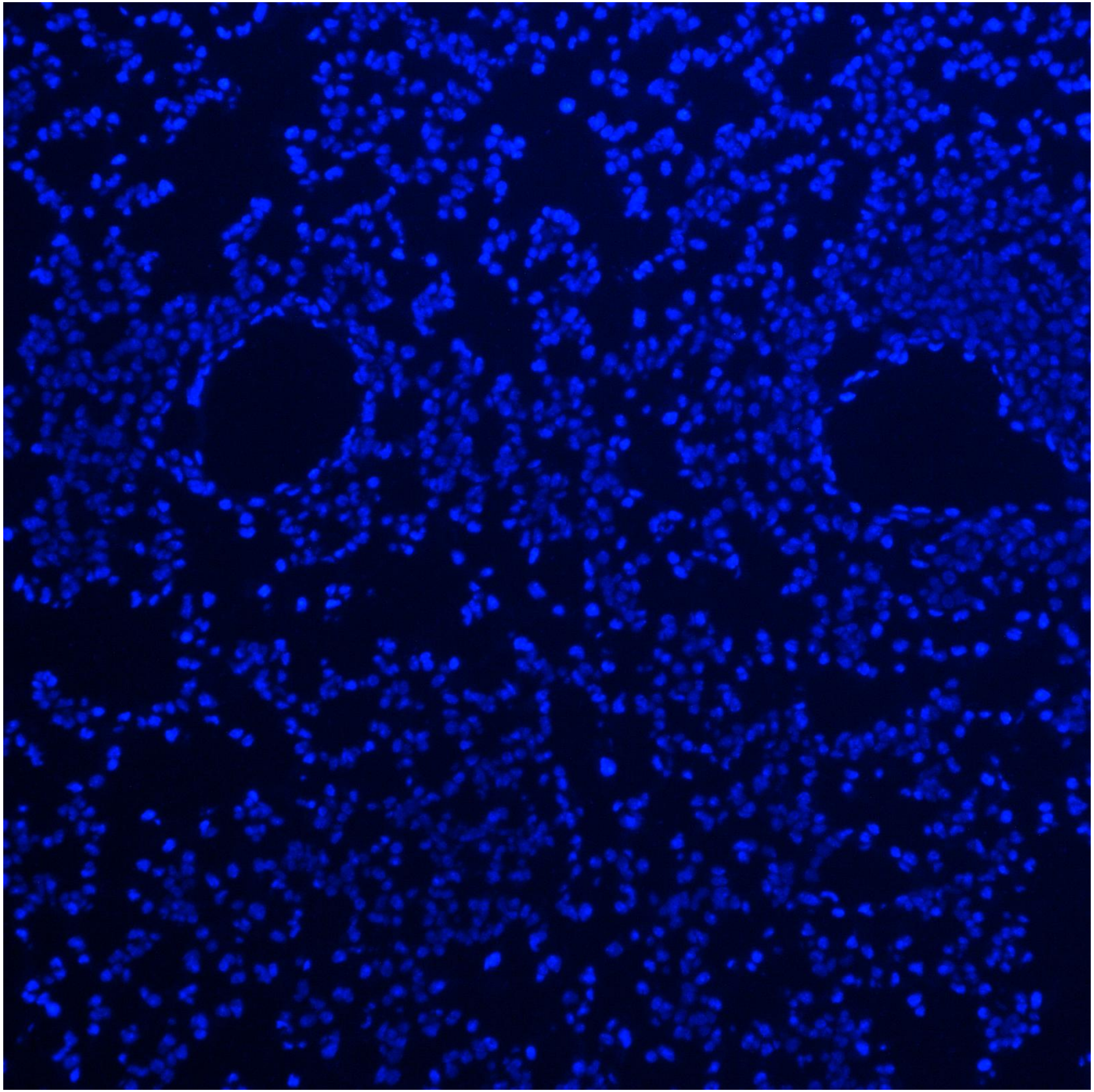

iri+LPS+apelin group Merge

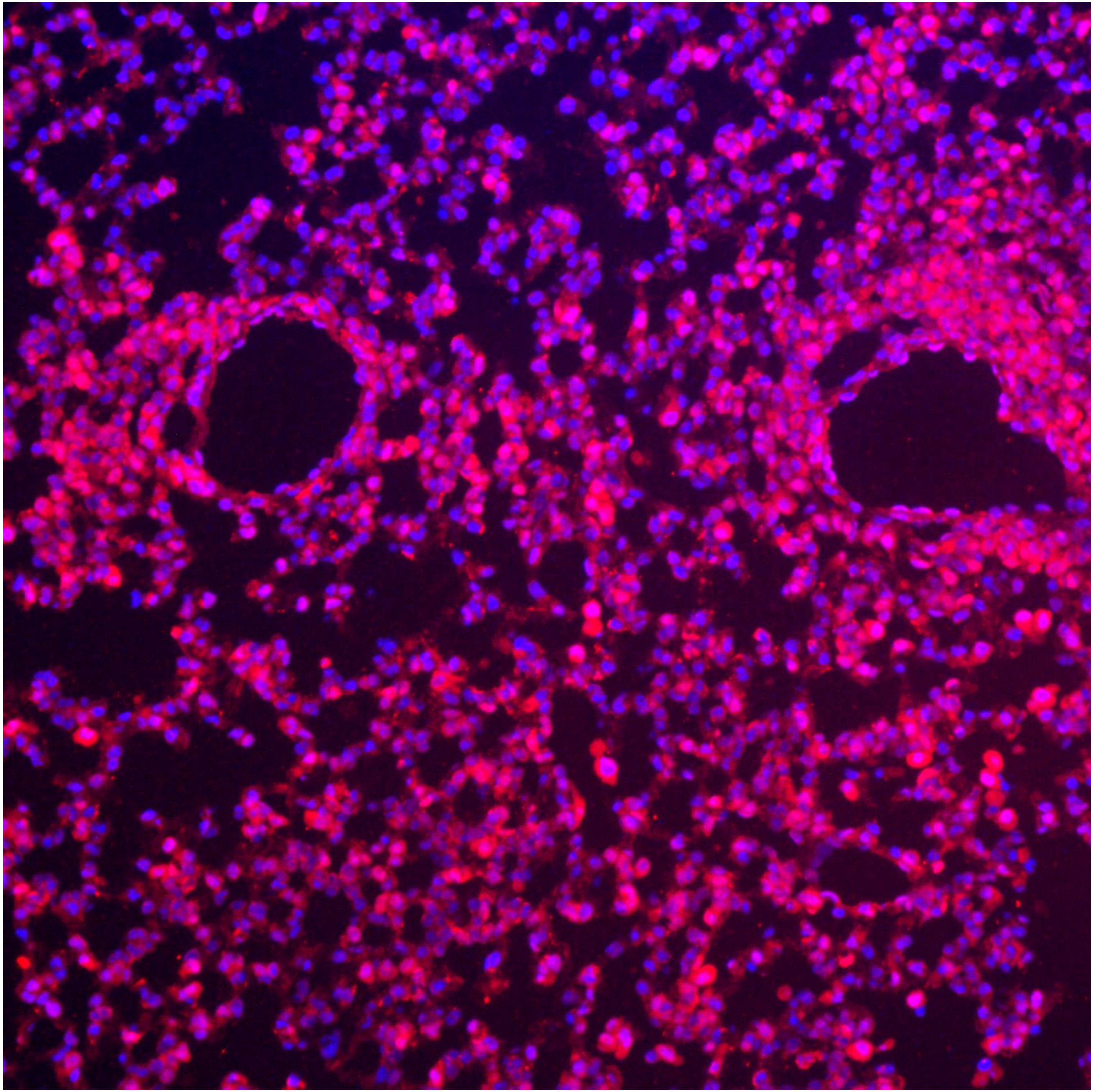

irinotecan group VE-cadherin

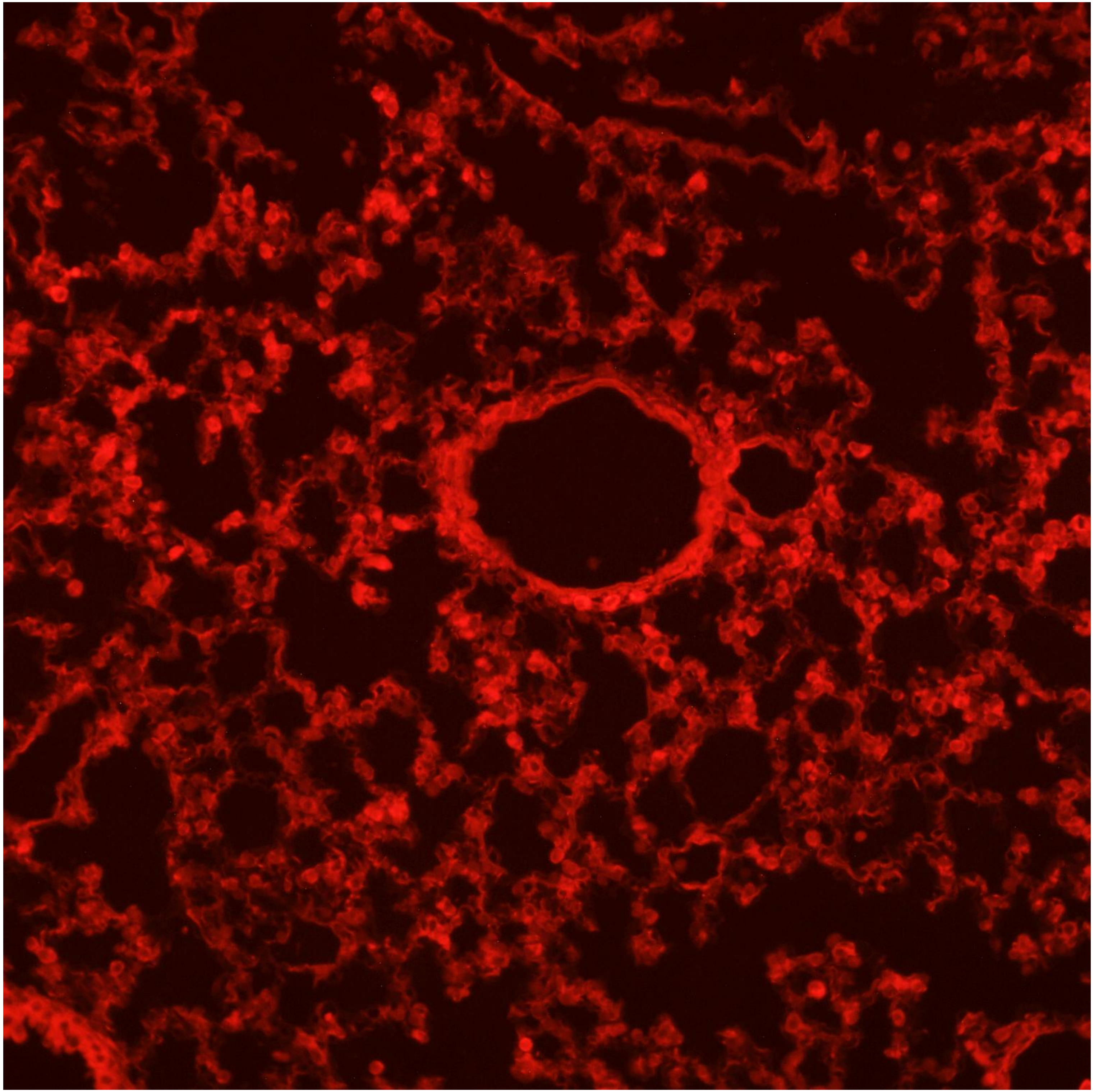

irinotecan group DAPI

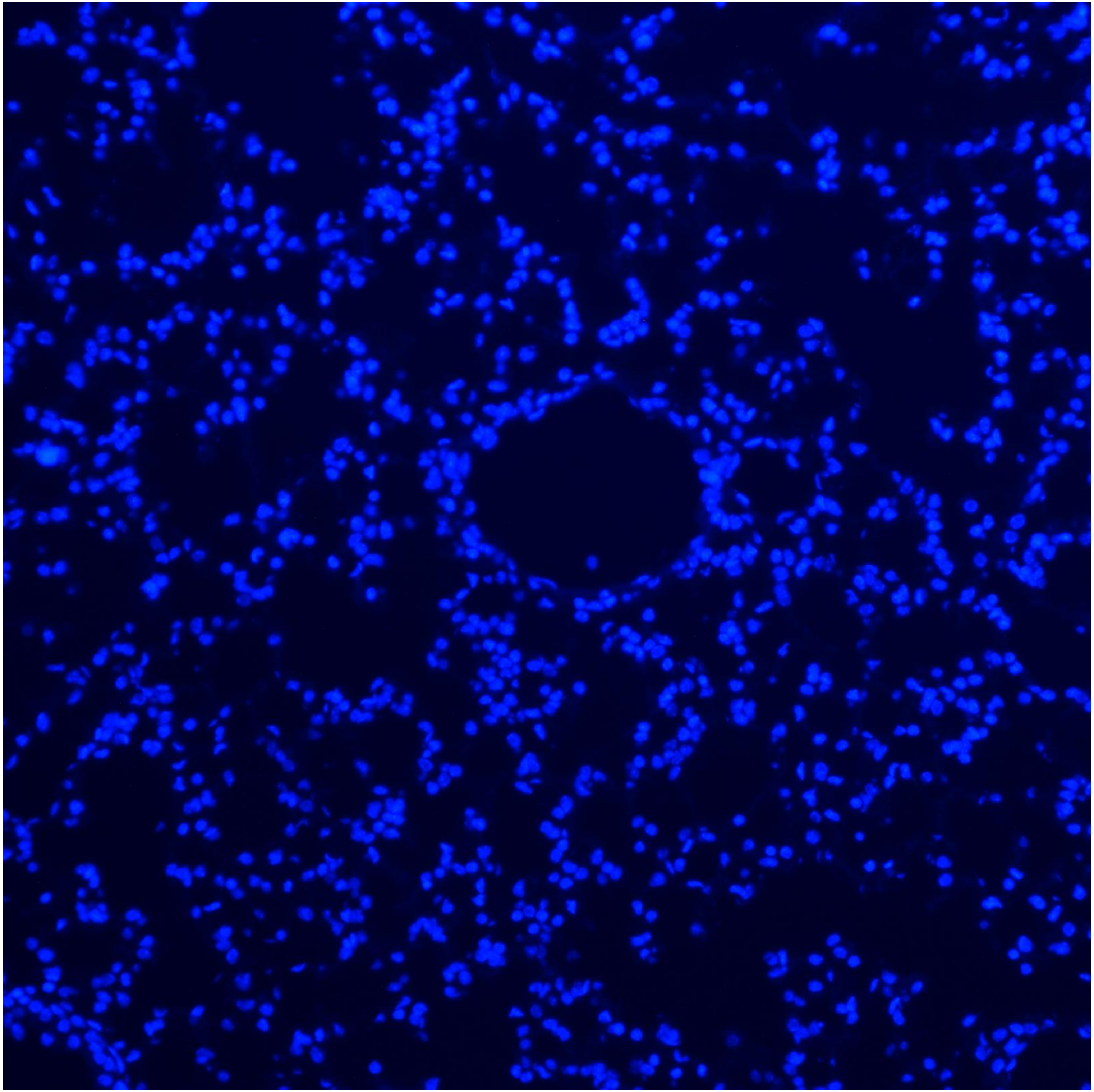

irinotecan group Merge

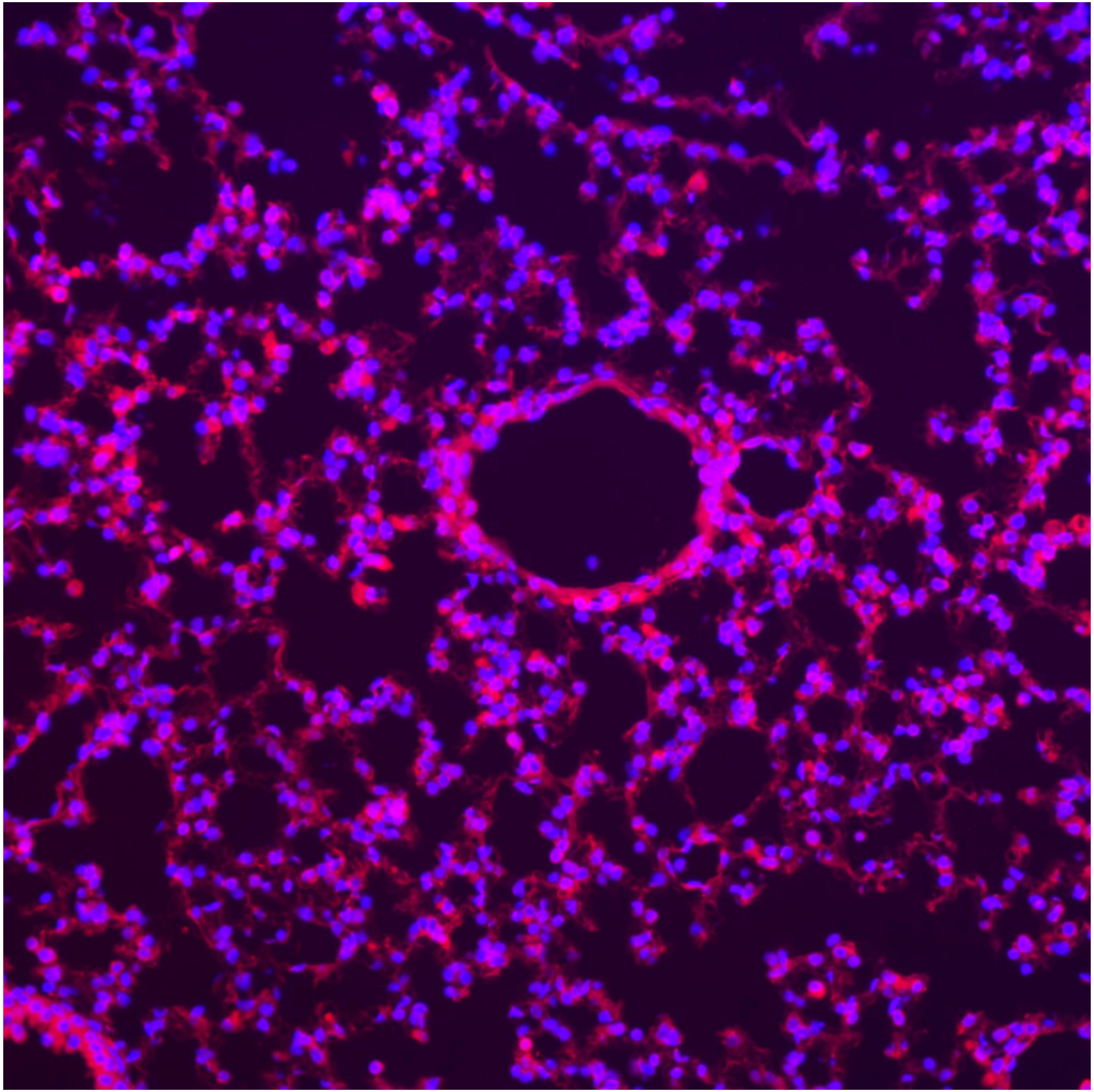

Supplement: Supplementary file 1 — Supplementary Information 1. [file 41598_2023_27889_MOESM1_ESM.pdf]
