# Supplementary material for: Effect and mechanism of apelin on lipopolysaccharide induced acute pulmonary vascular endothelial barrier dysfunction
Source: Sci Rep. 2023 Jan 27;13:1560. doi: 10.1038/s41598-023-27889-6 (PMC9883263; doi:10.1038/s41598-023-27889-6)

Figure 2A Fli-1

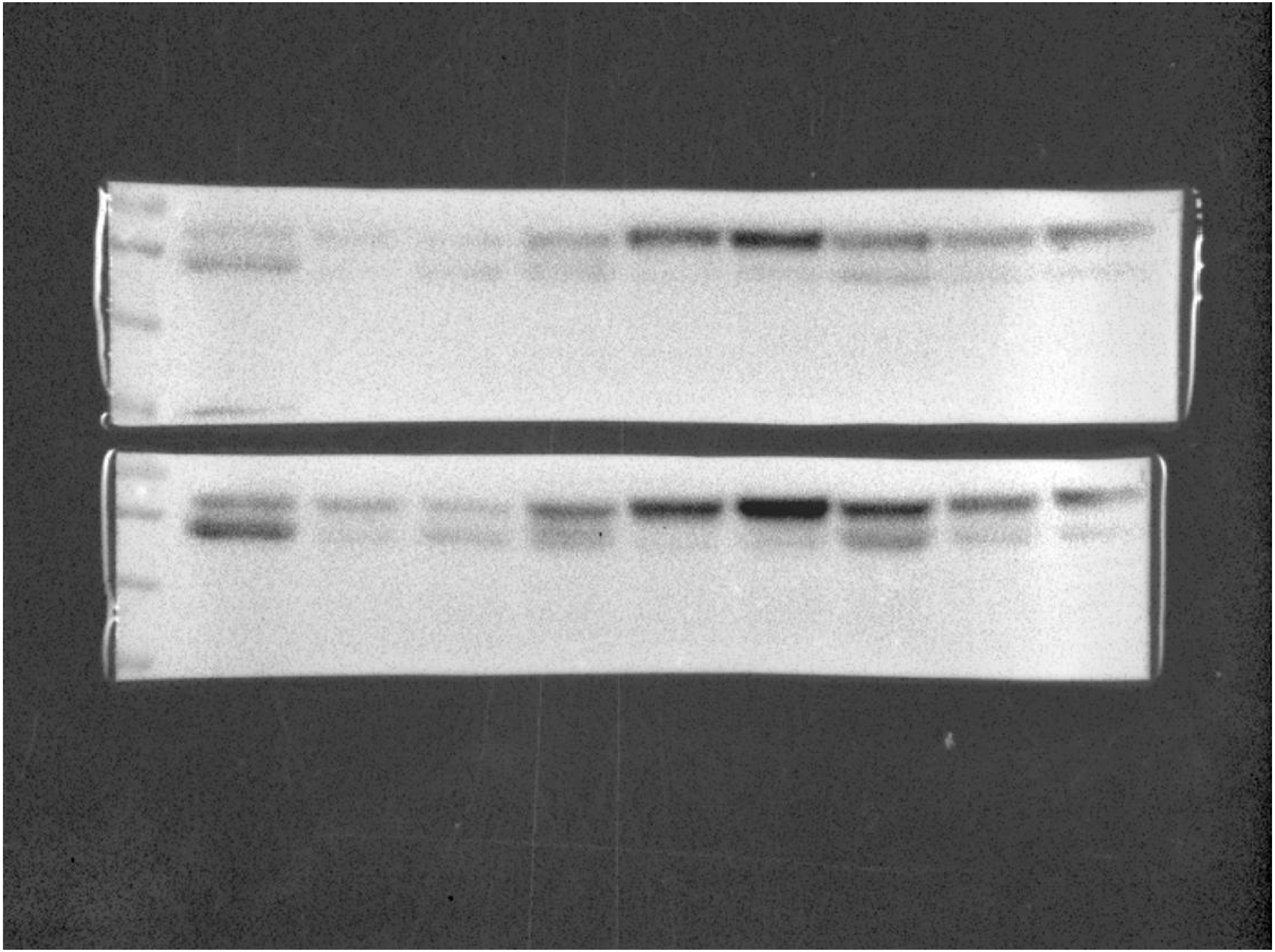

Figure 2A SRC

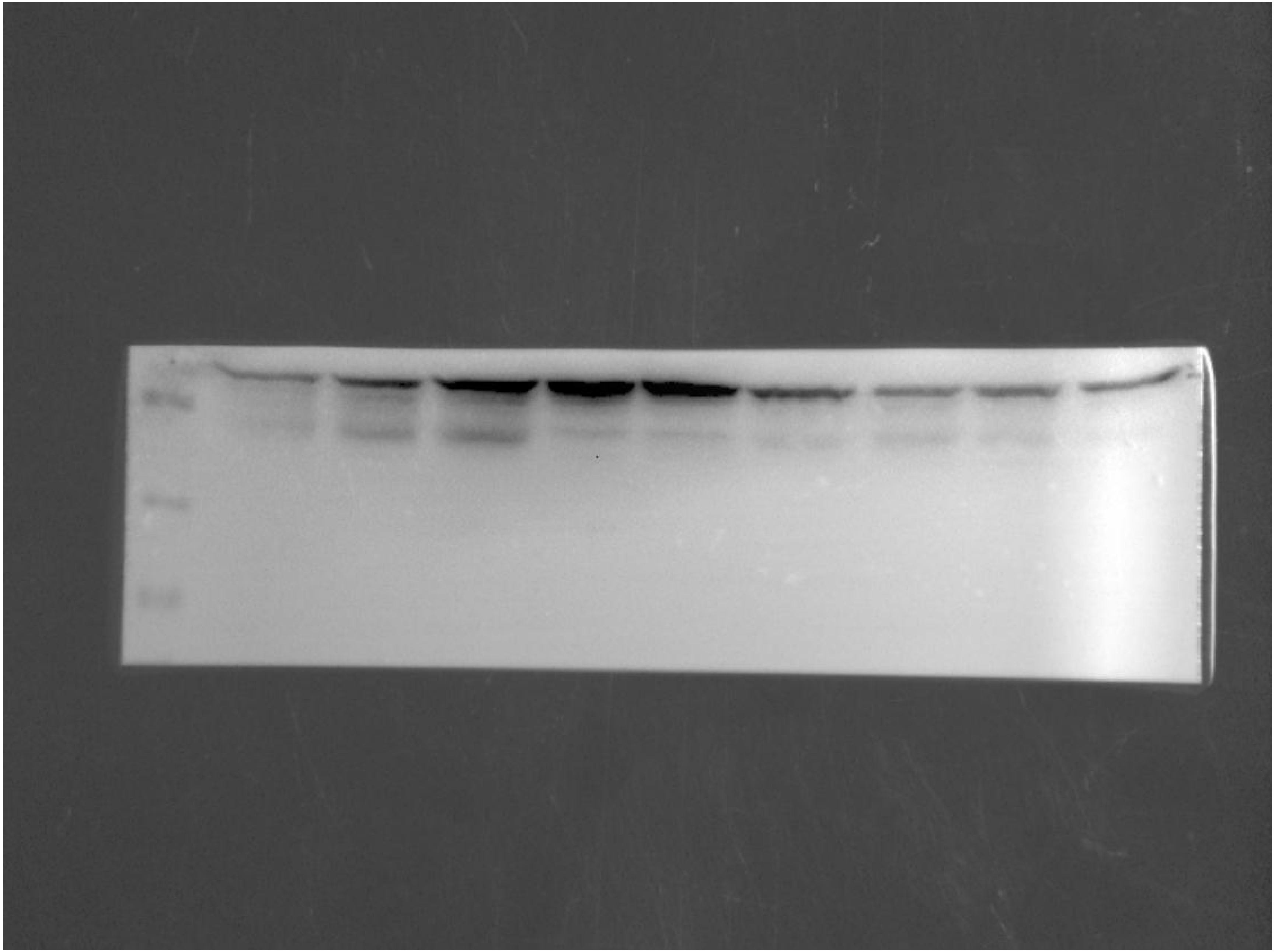

Figure 2A and Figure 5A GAPDH

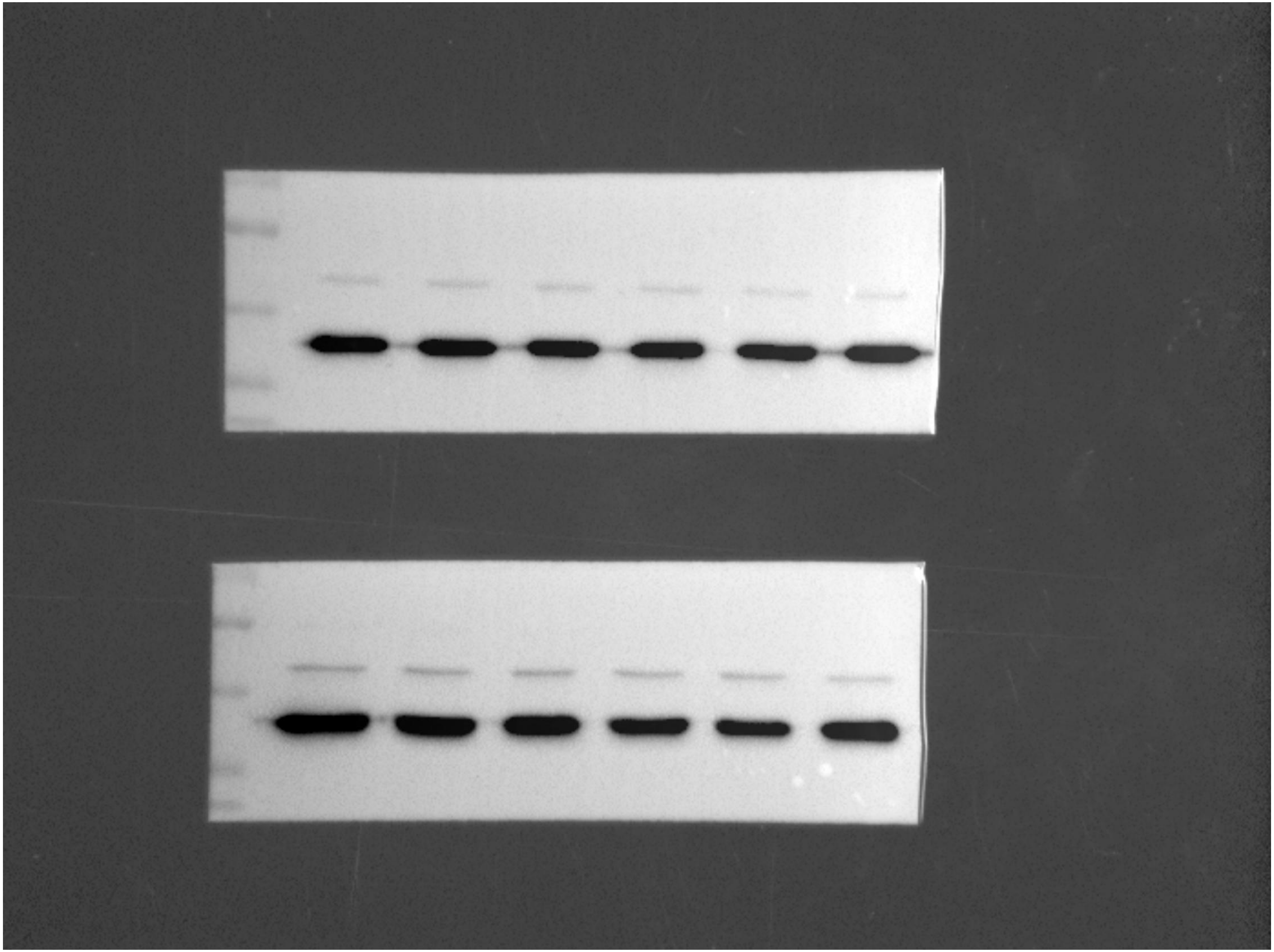

Figure 4A Fli-1

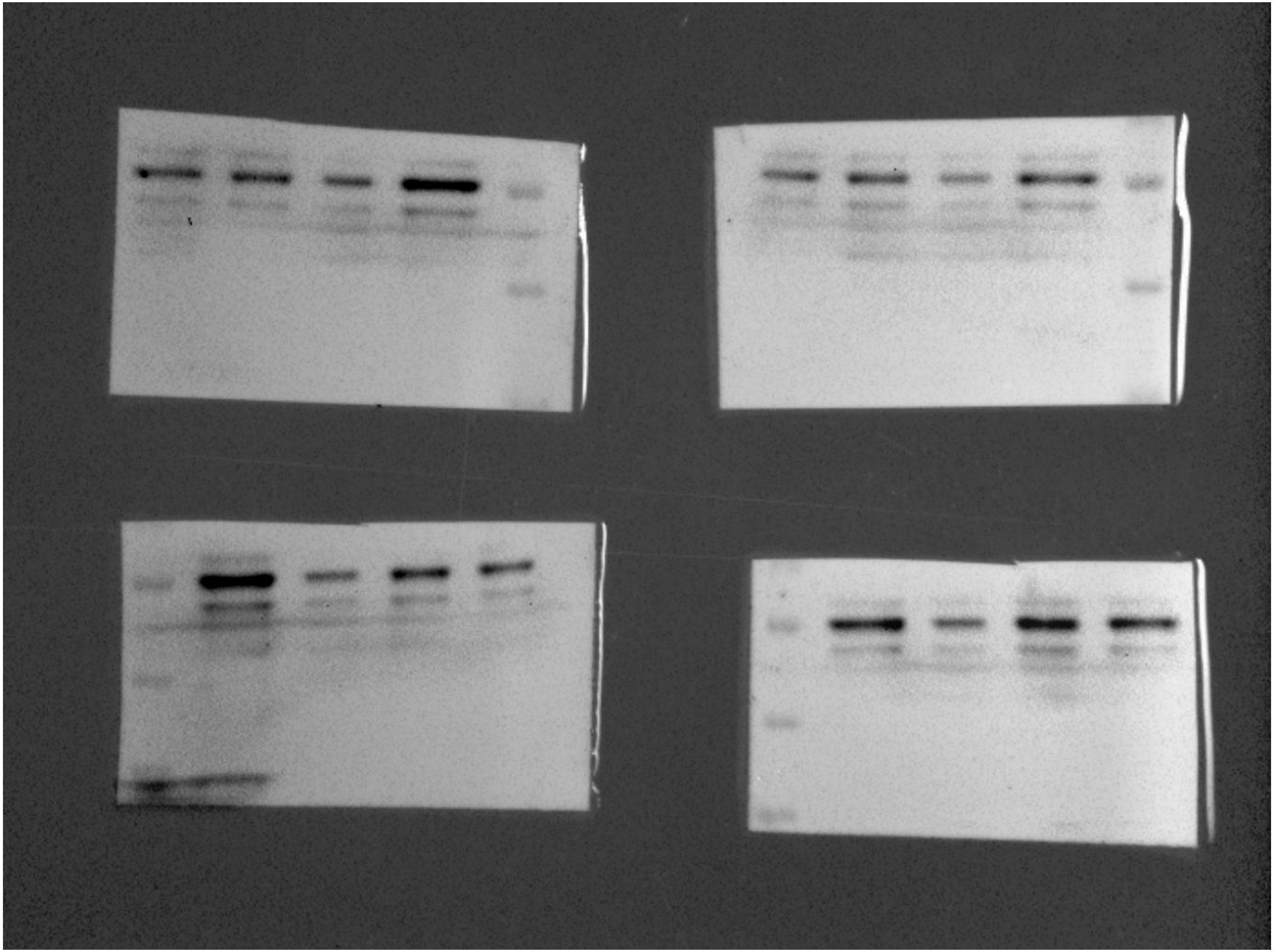

Figure 4A VEGF

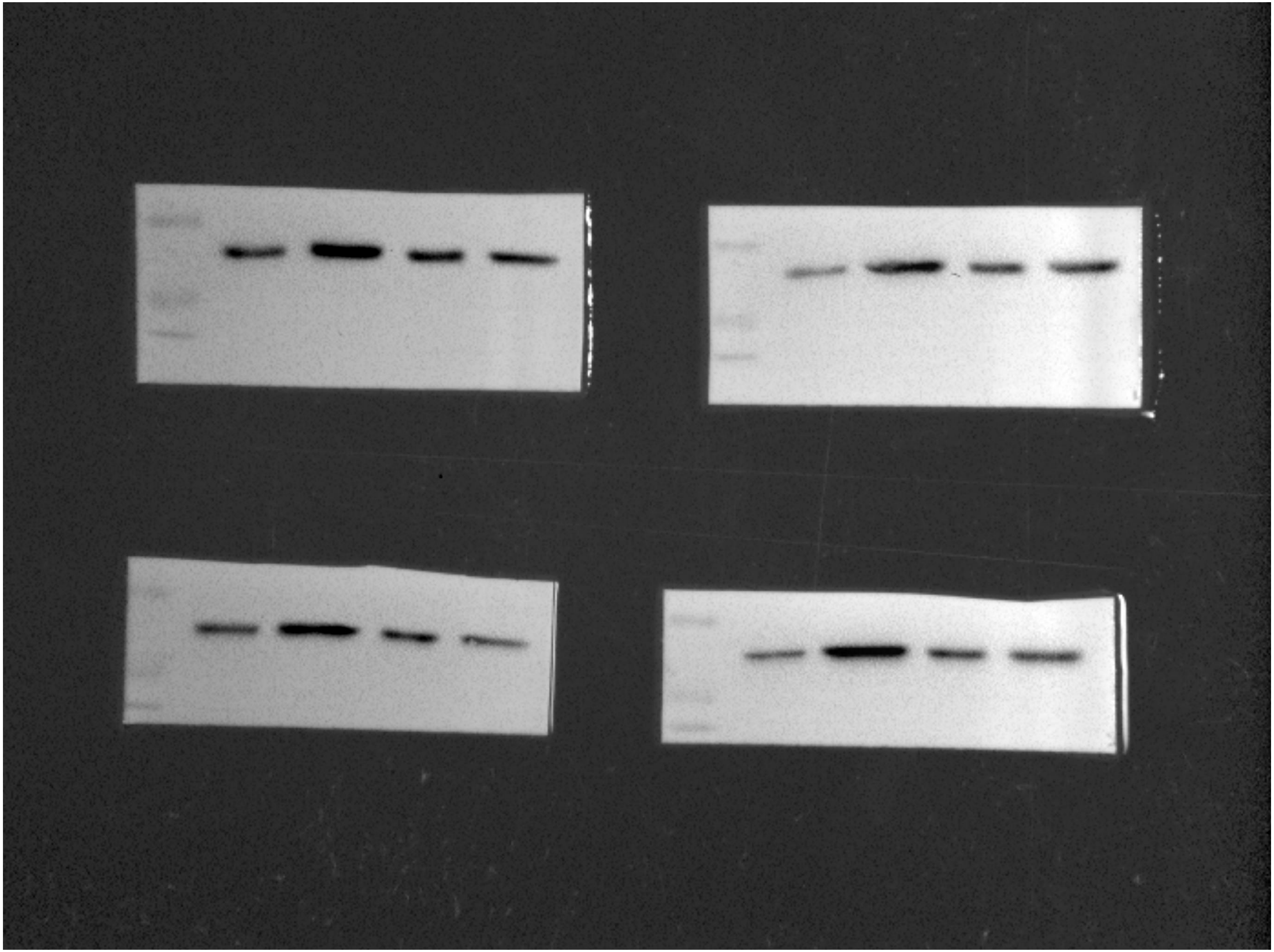

Figure 4A VEGF-R

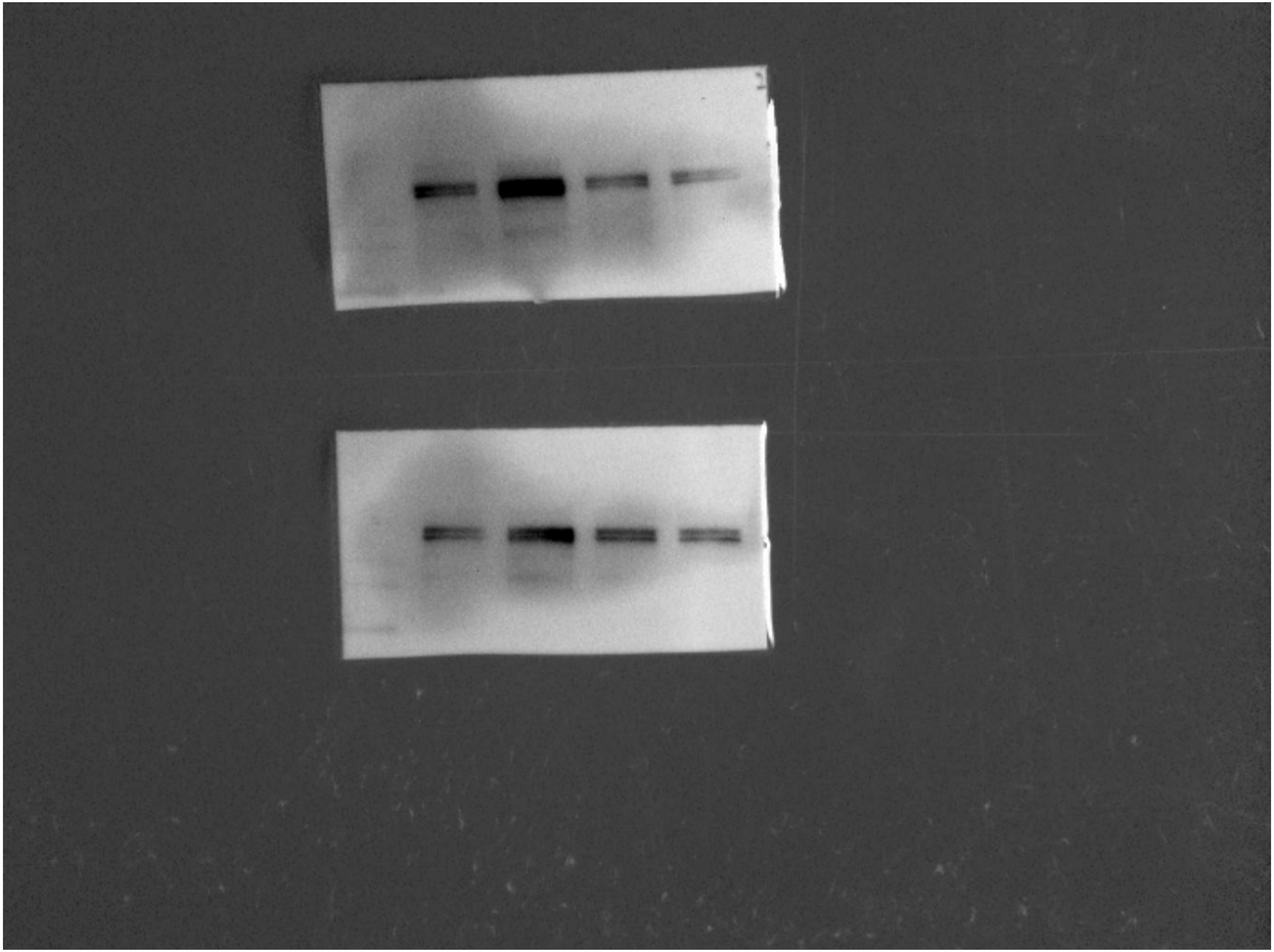

Figure 4A SRC

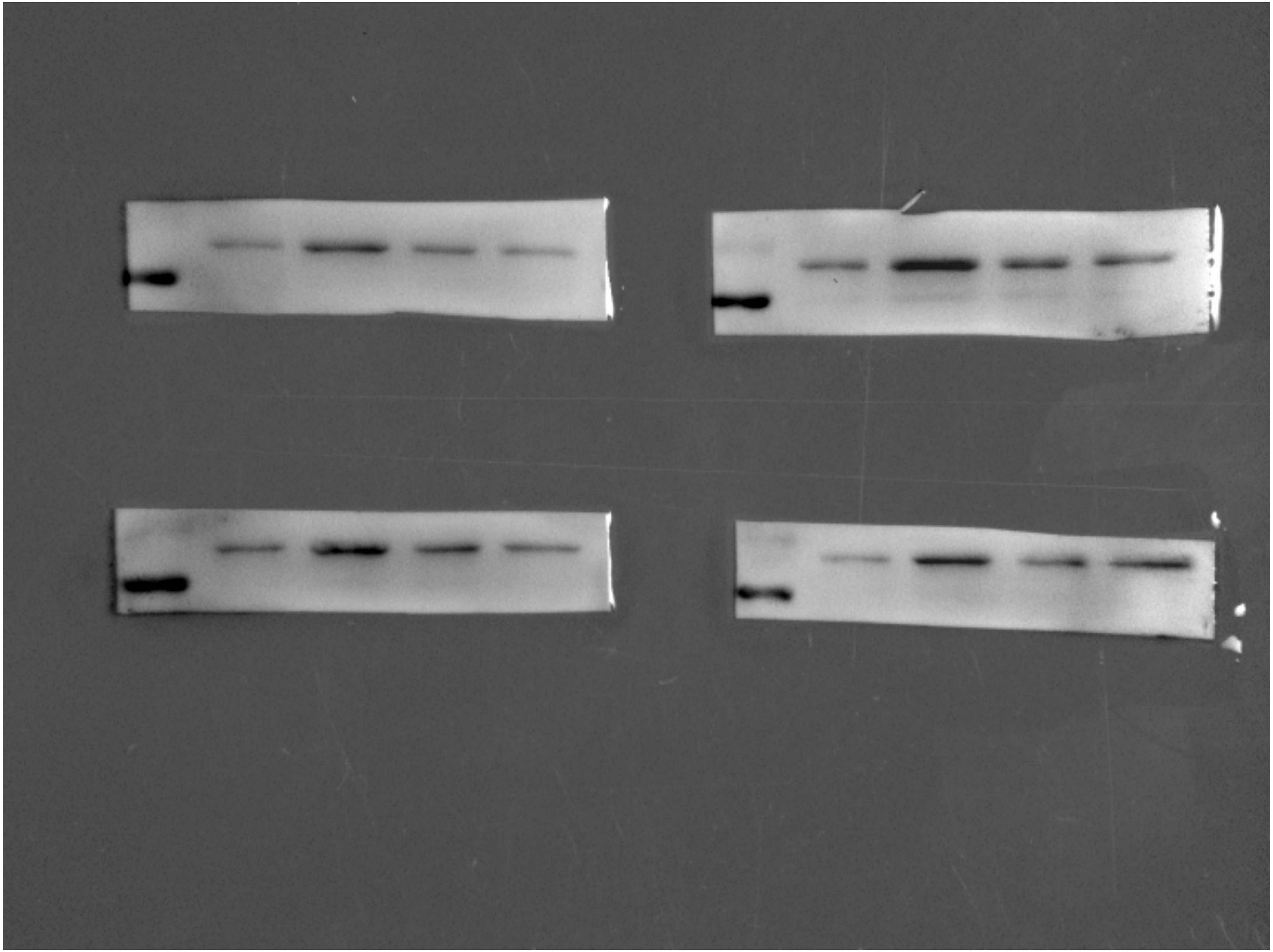

Figure 4A p-SRC

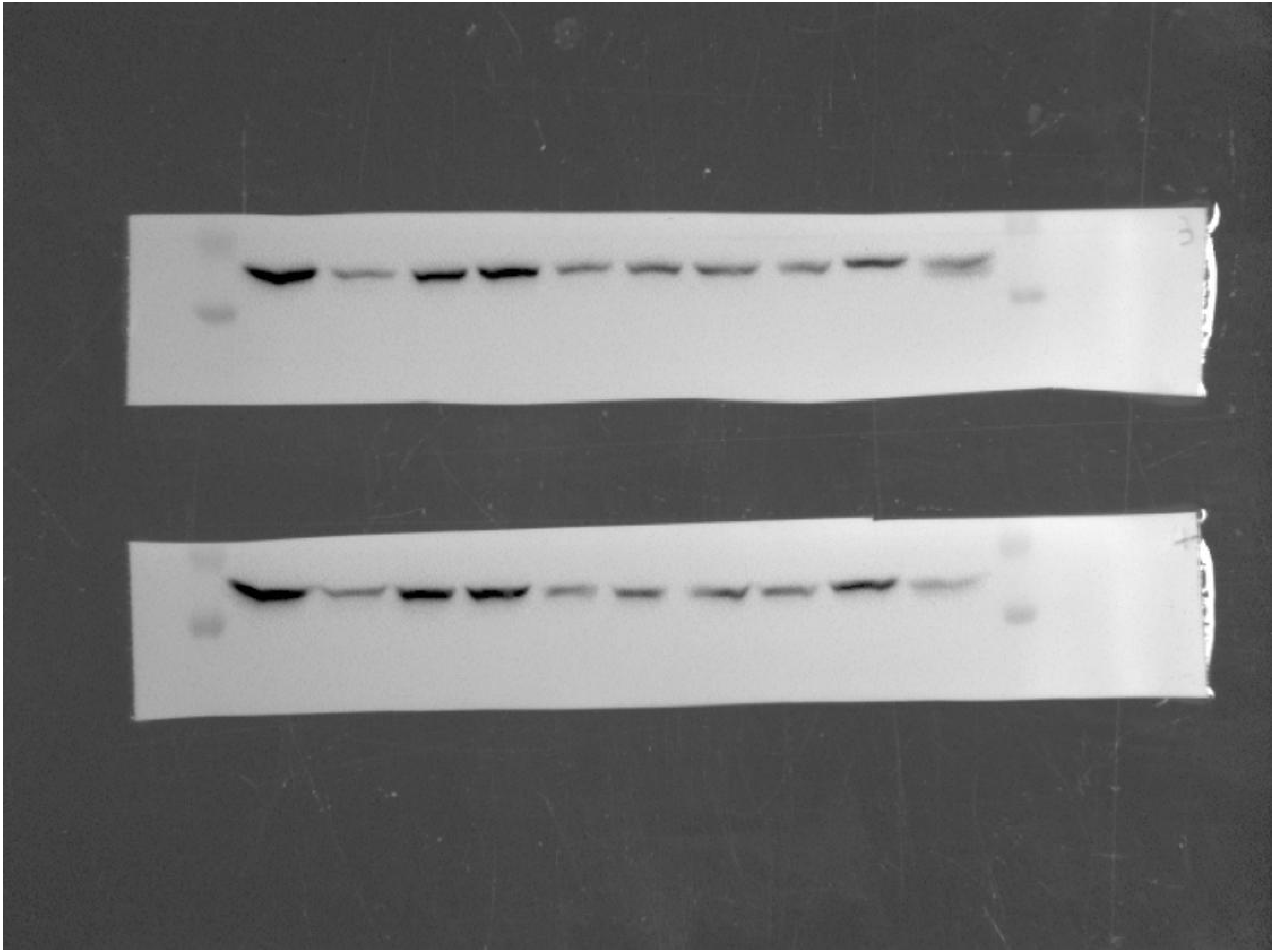

Figure 4A VE-cadherin

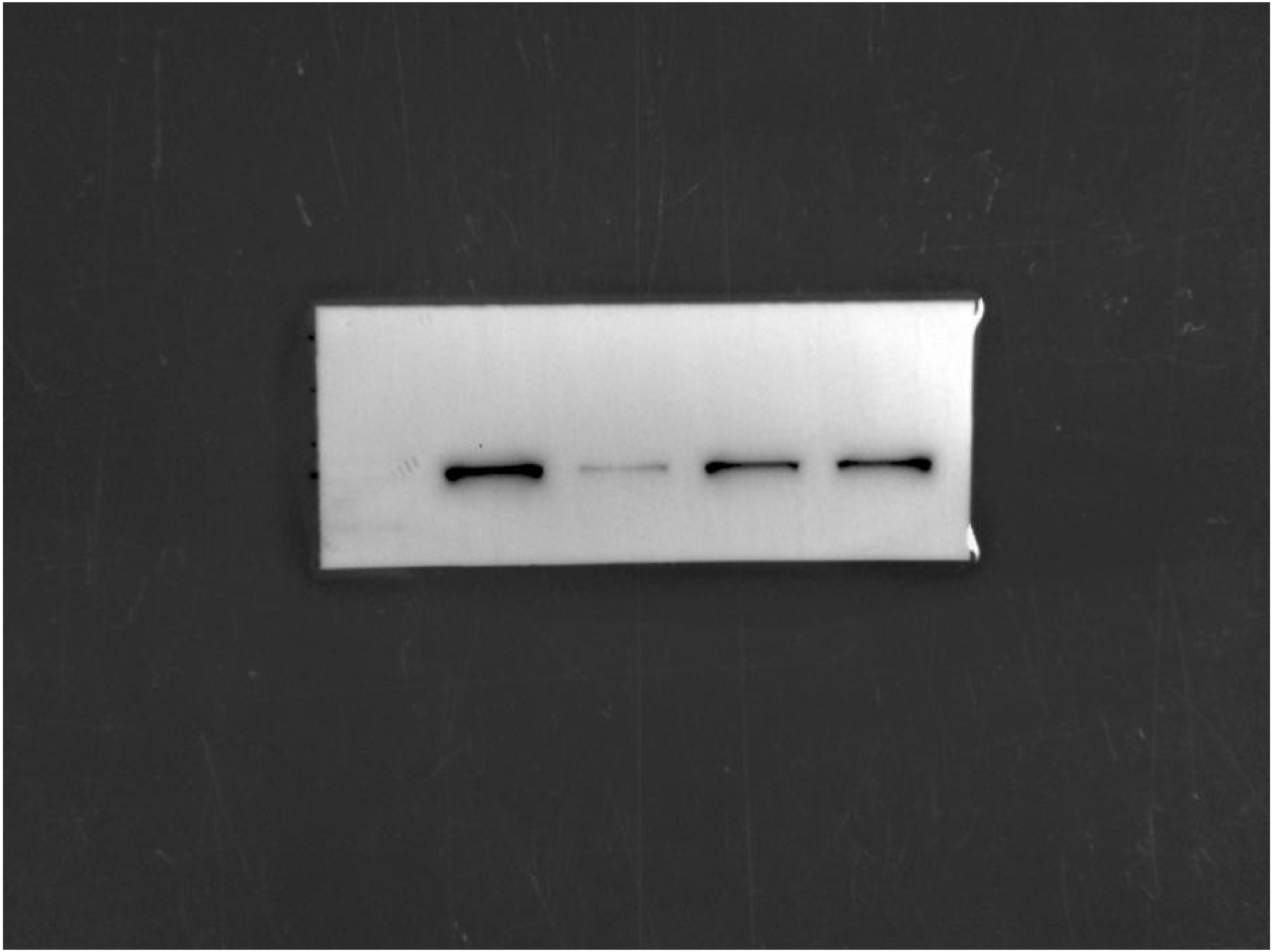

Figure 4A p-VE-cadherin

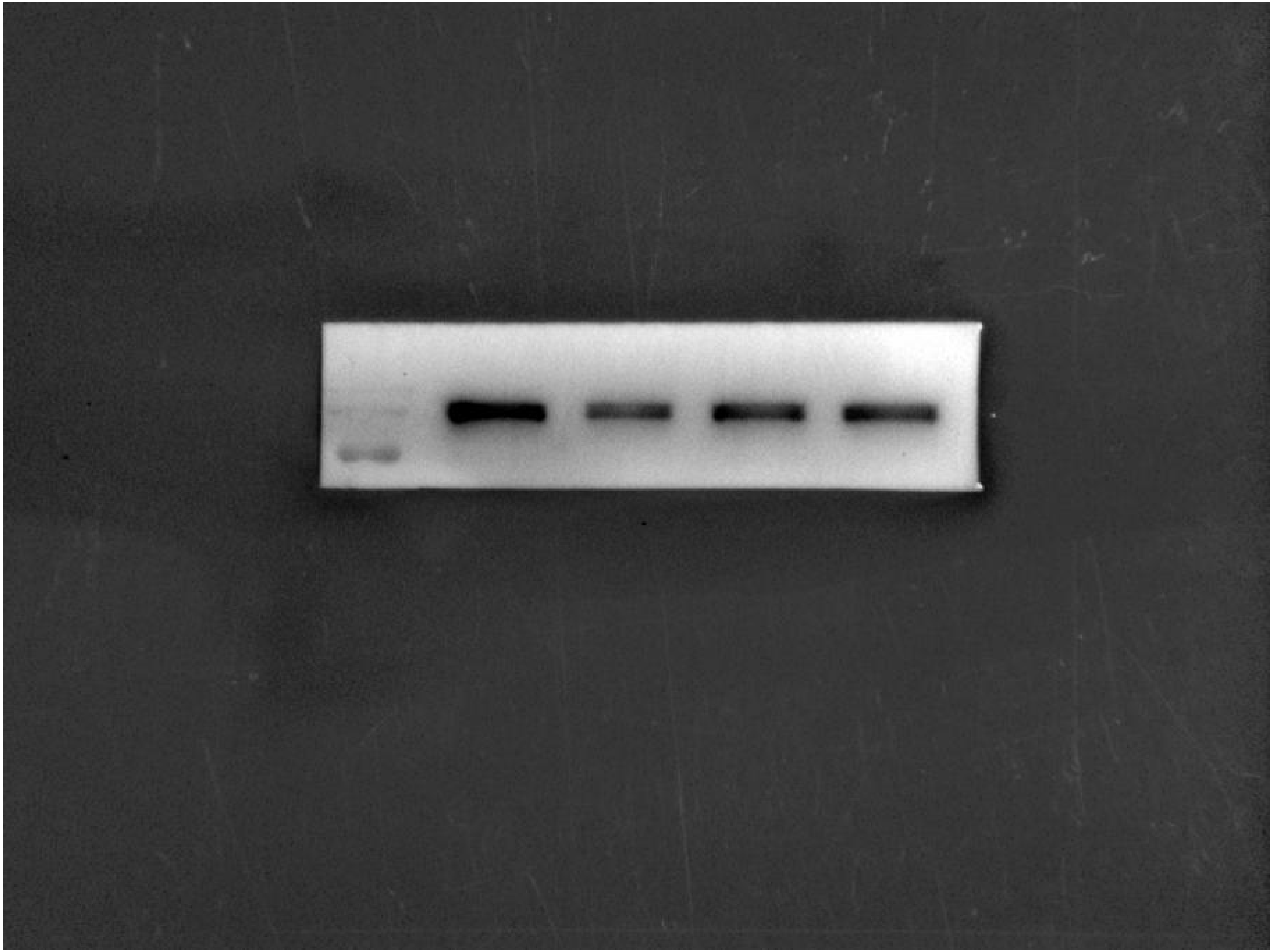

Figure 4A β-catenin

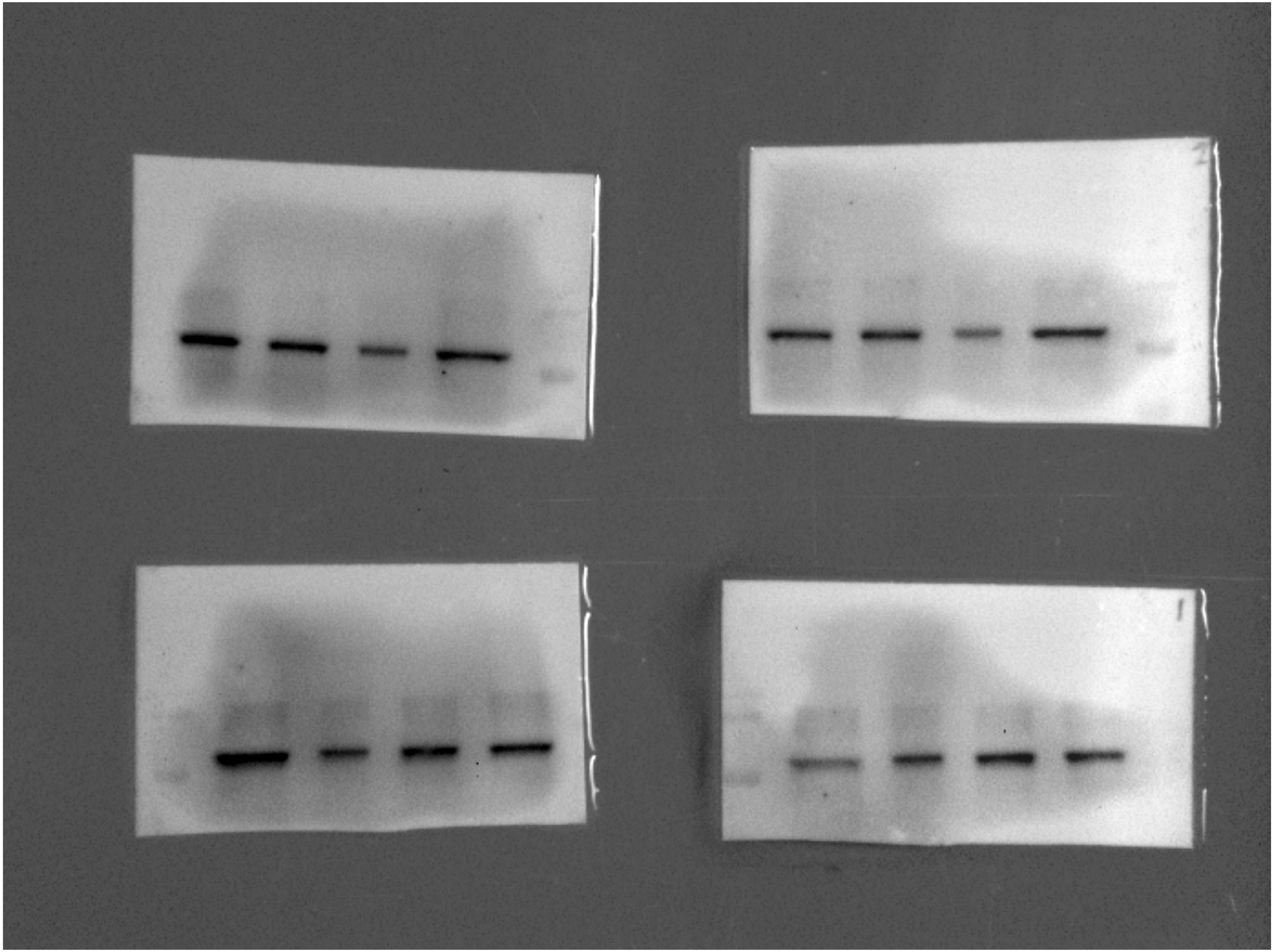

Figure 4A APJ

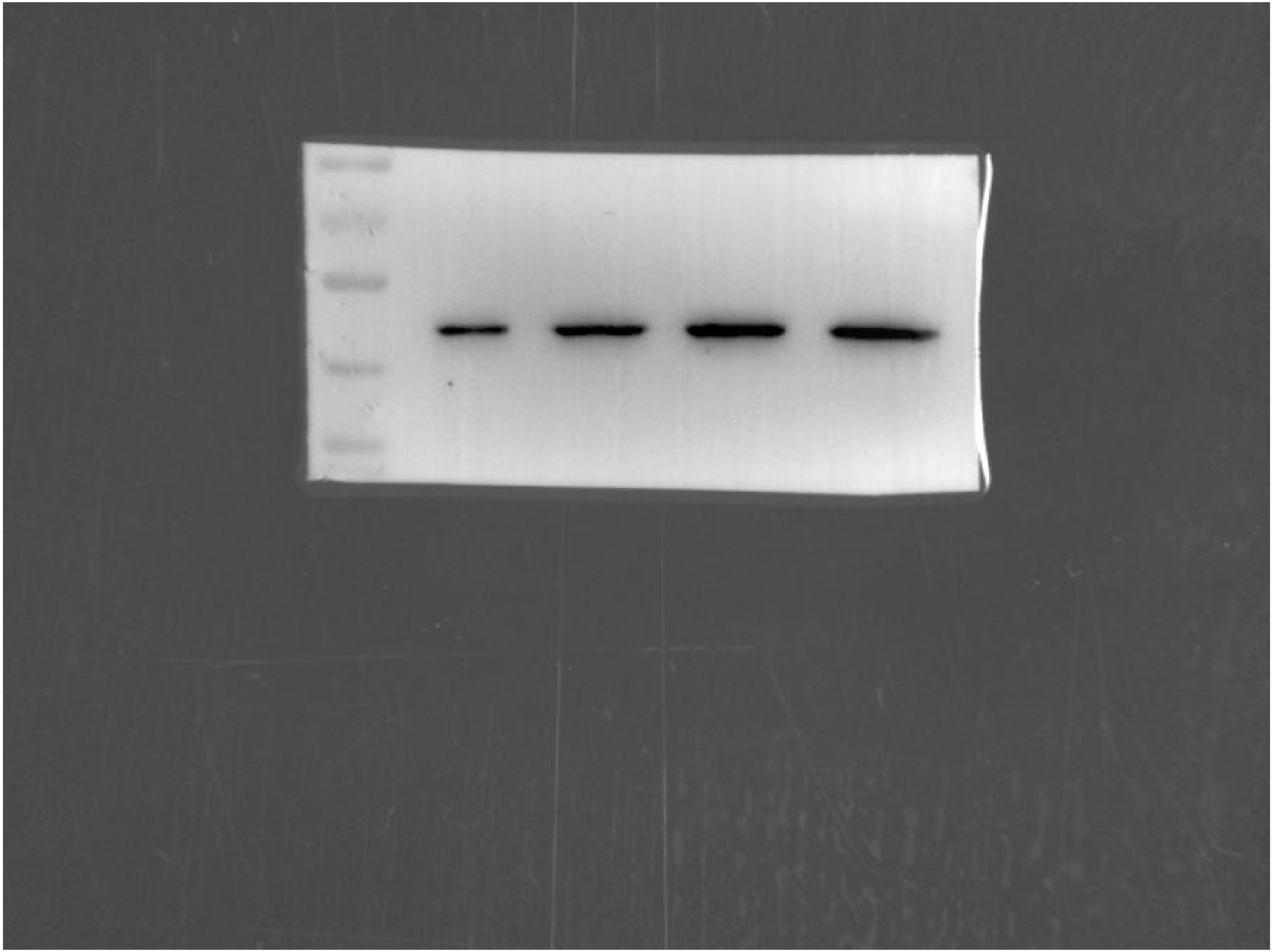

Figure 4A GAPDH

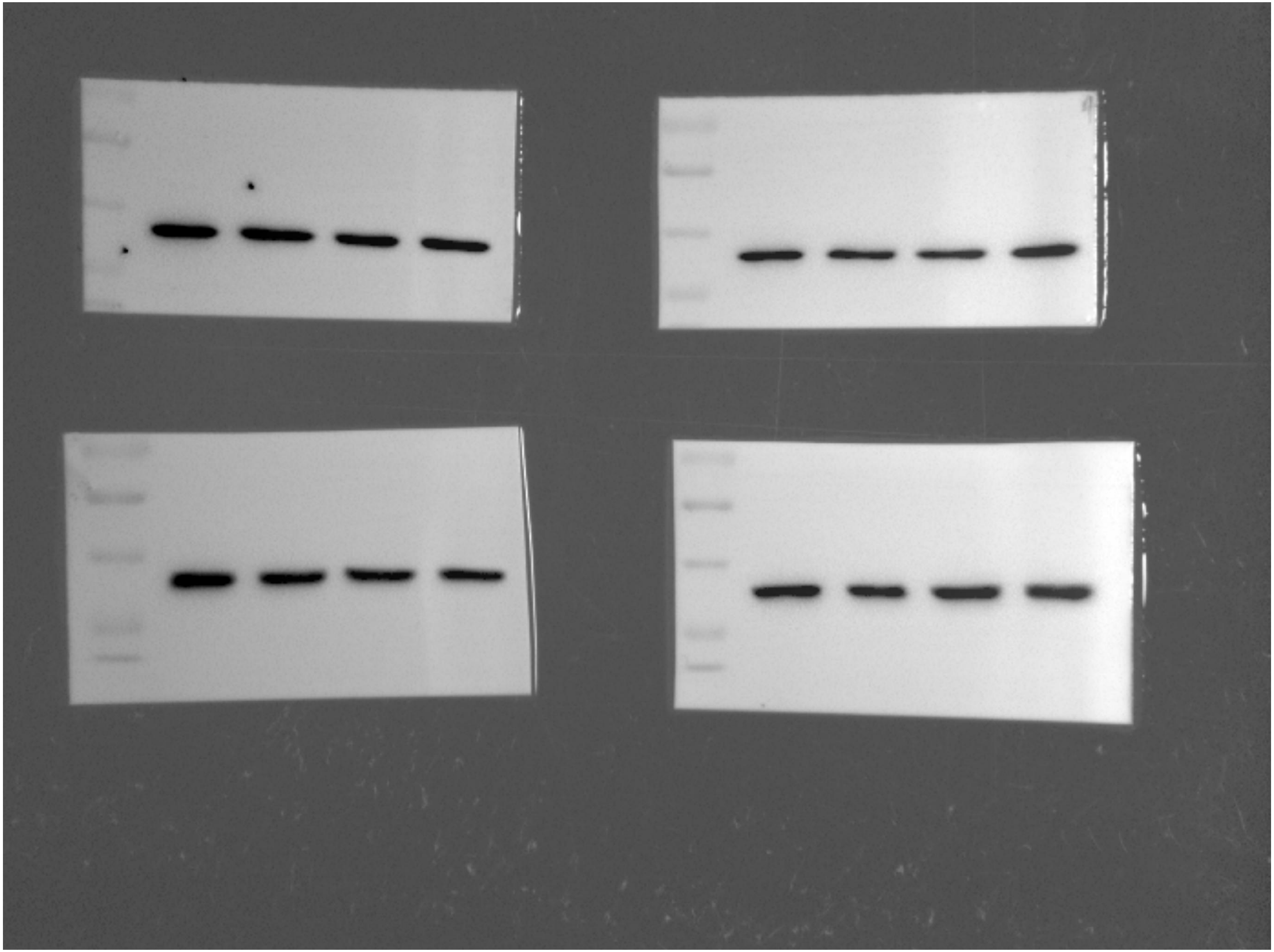

Figure 5A Fli-1

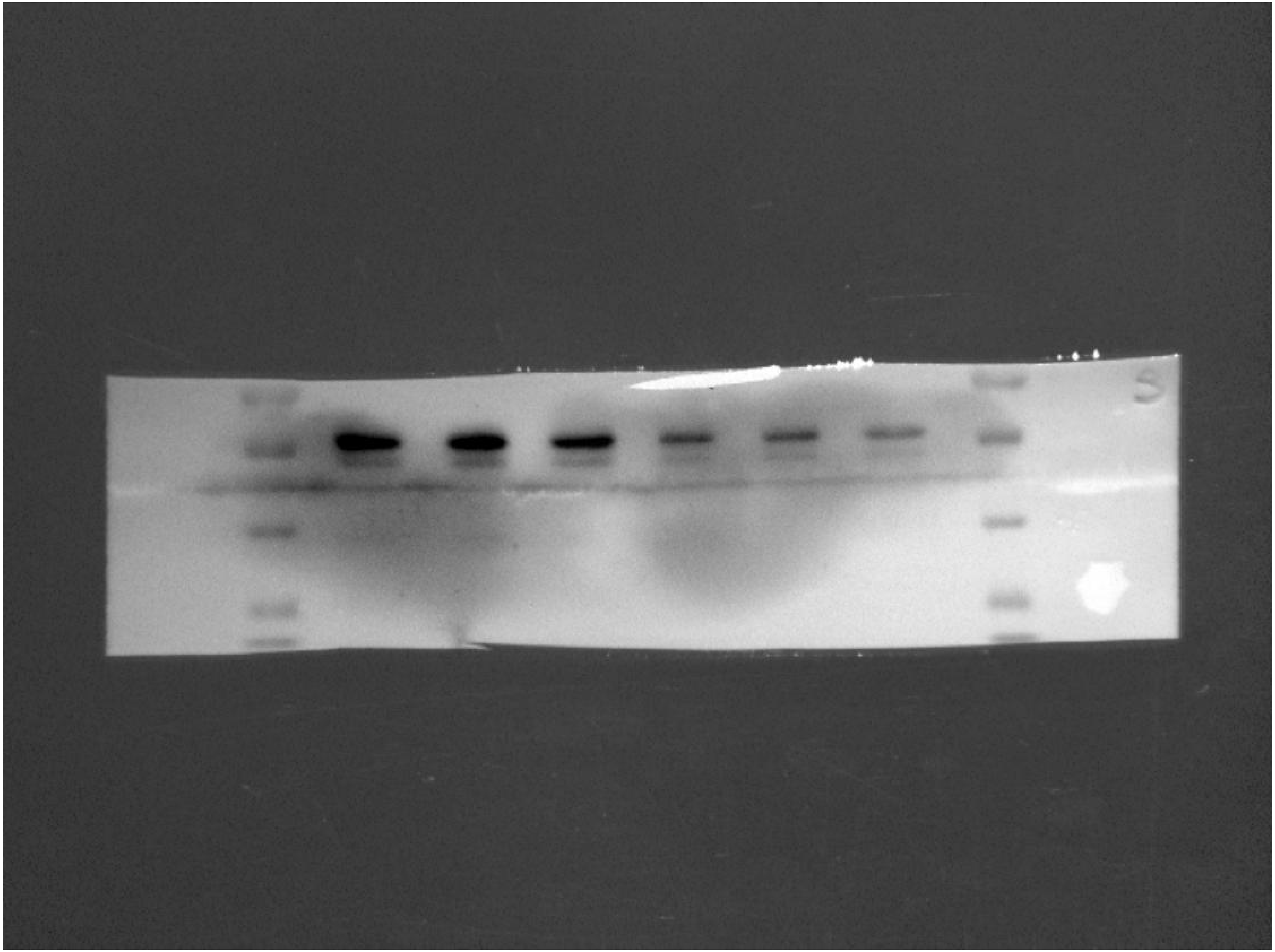

Figure 6A Fli-1

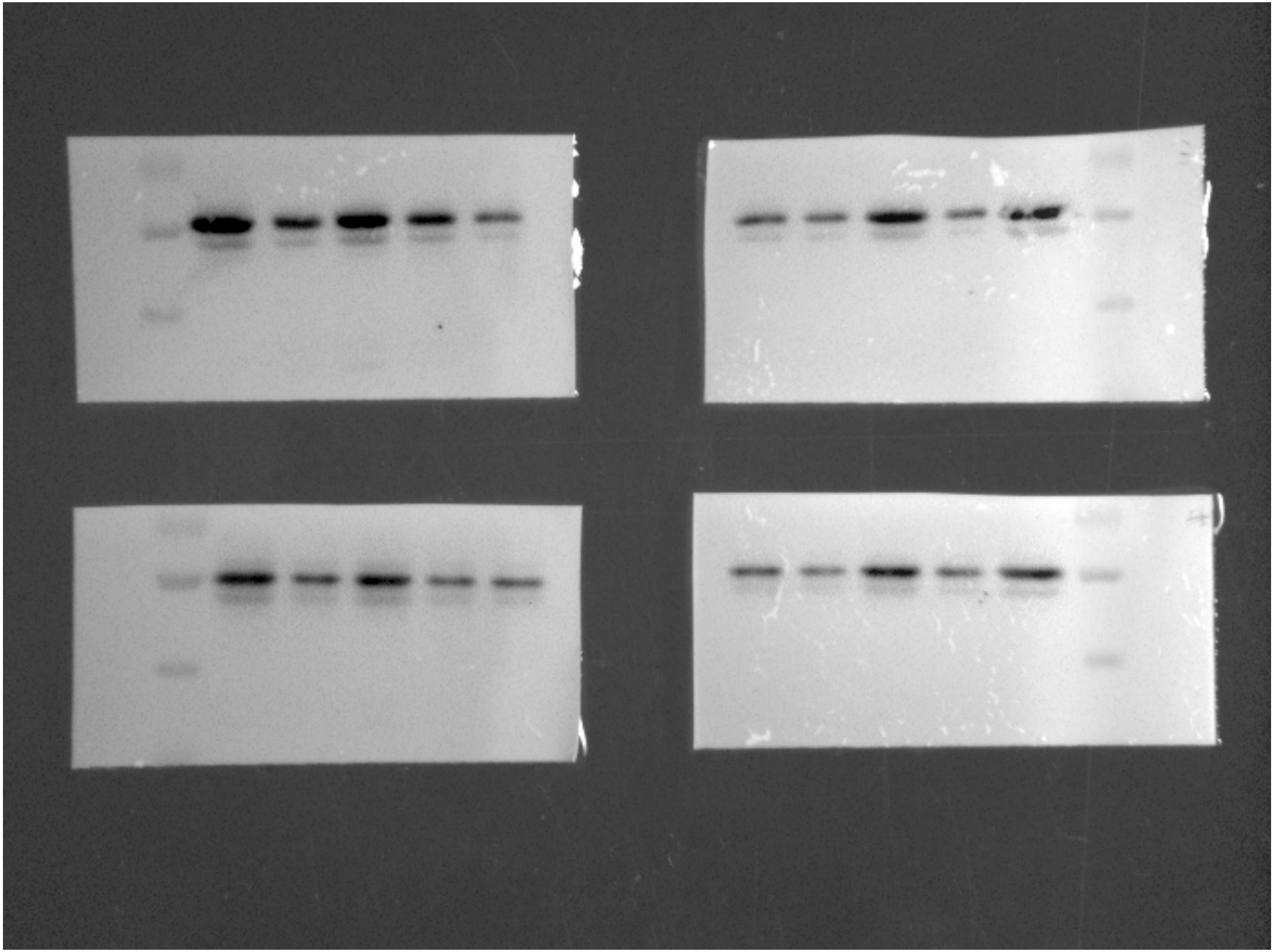

Figure 6A VEGF

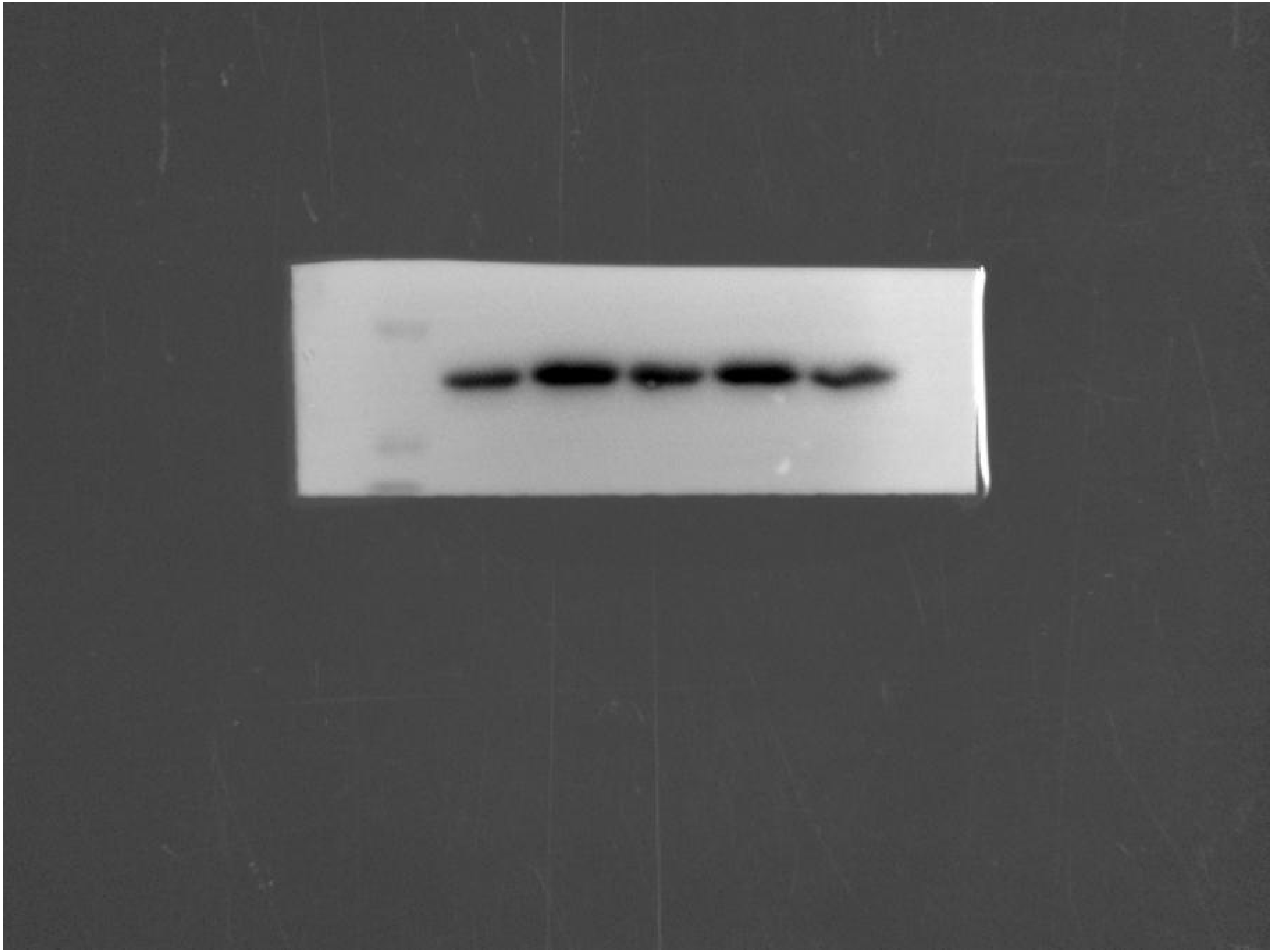

Figure 6A VEGF-R

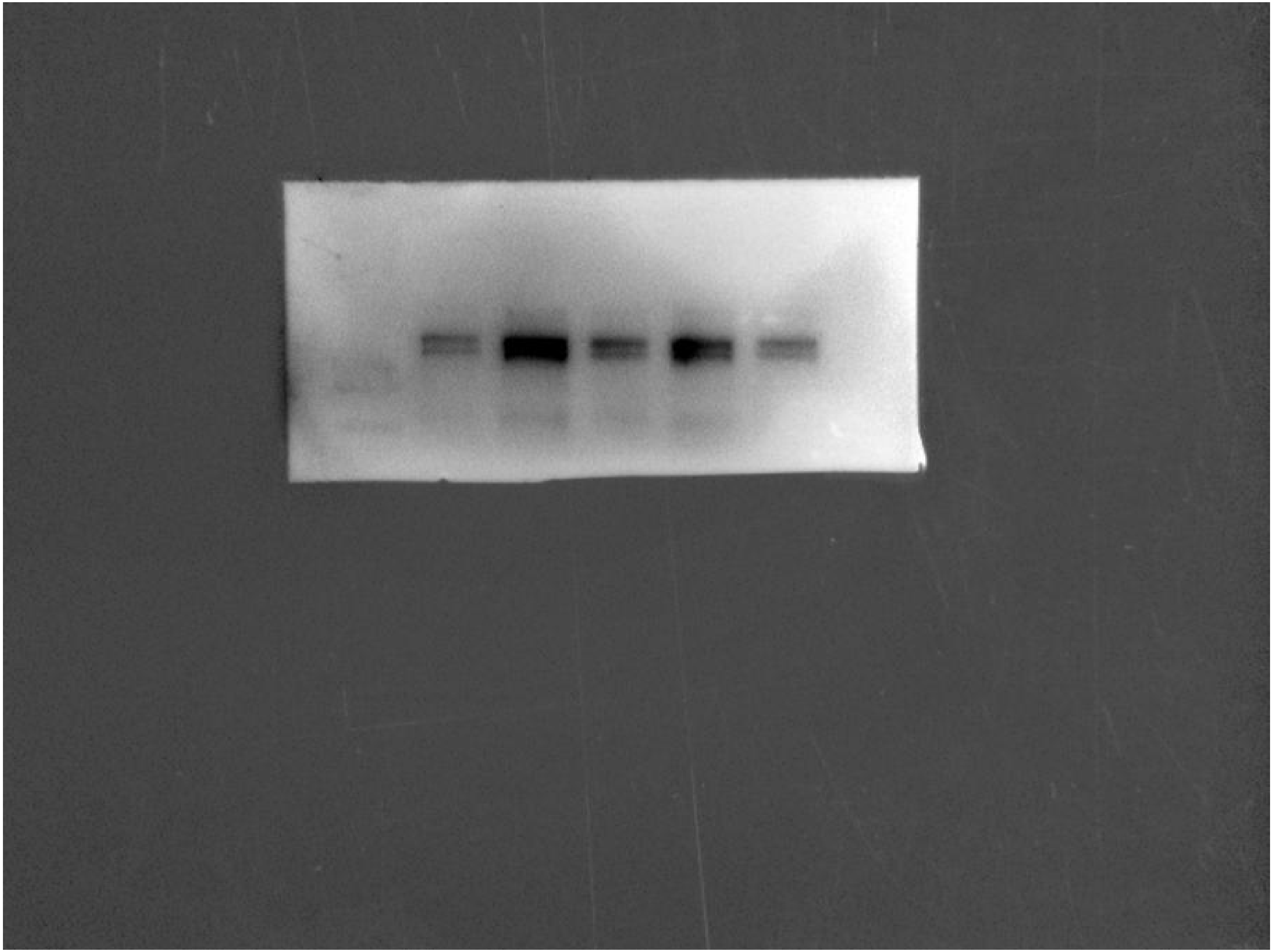

Figure 6A SRC

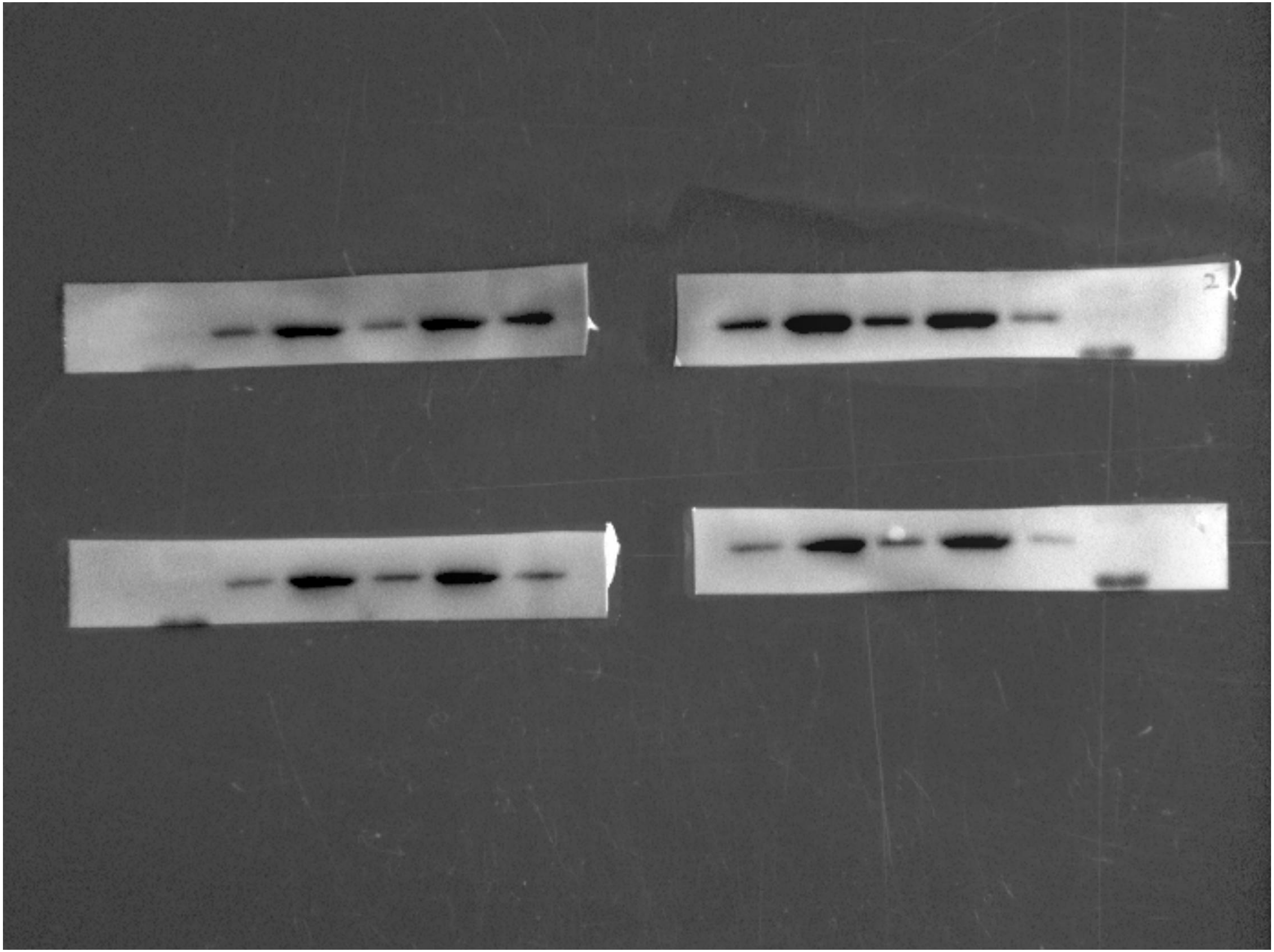

Figure 6A p-SRC

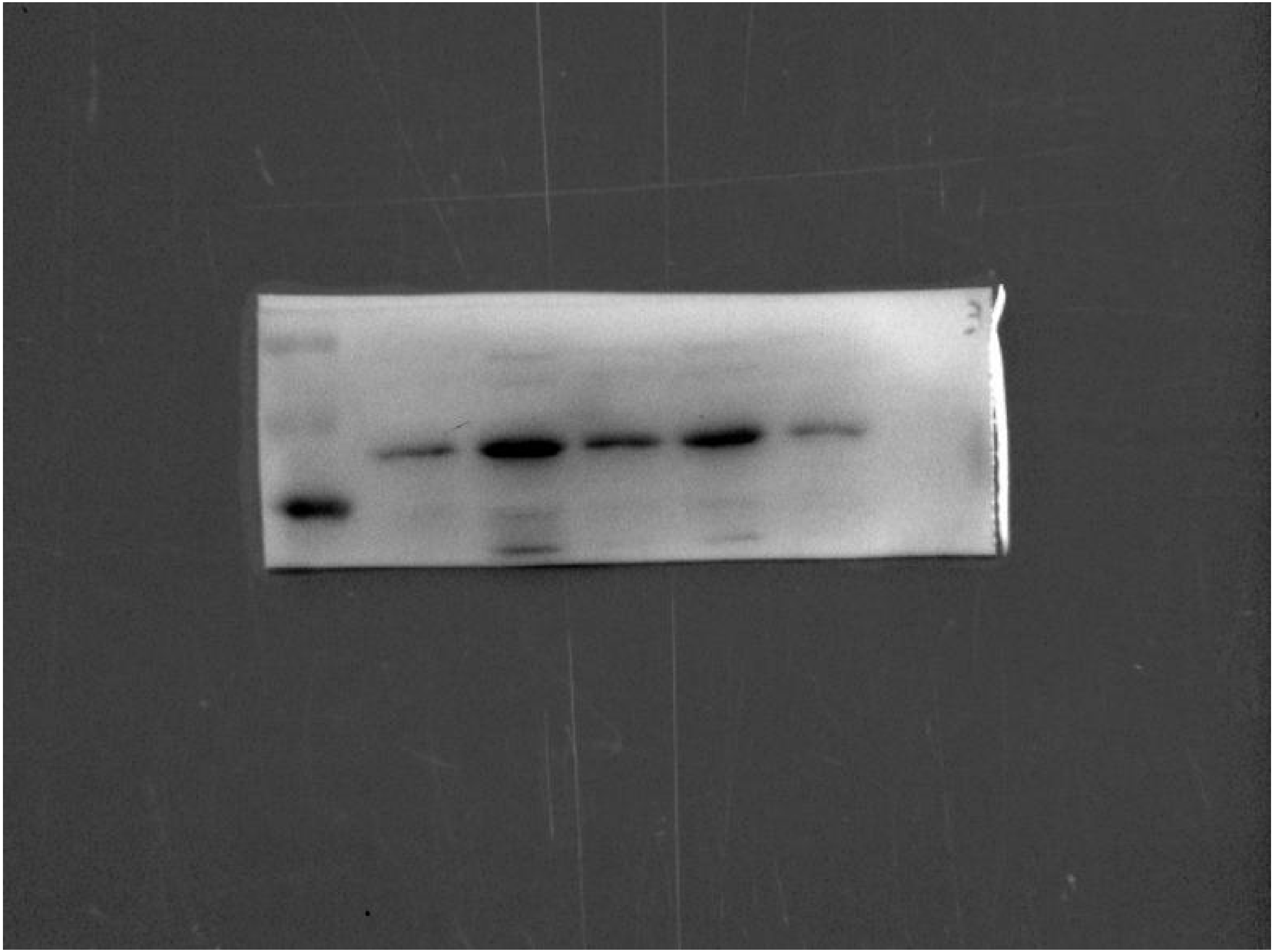

Figure 6A VE-cadherin

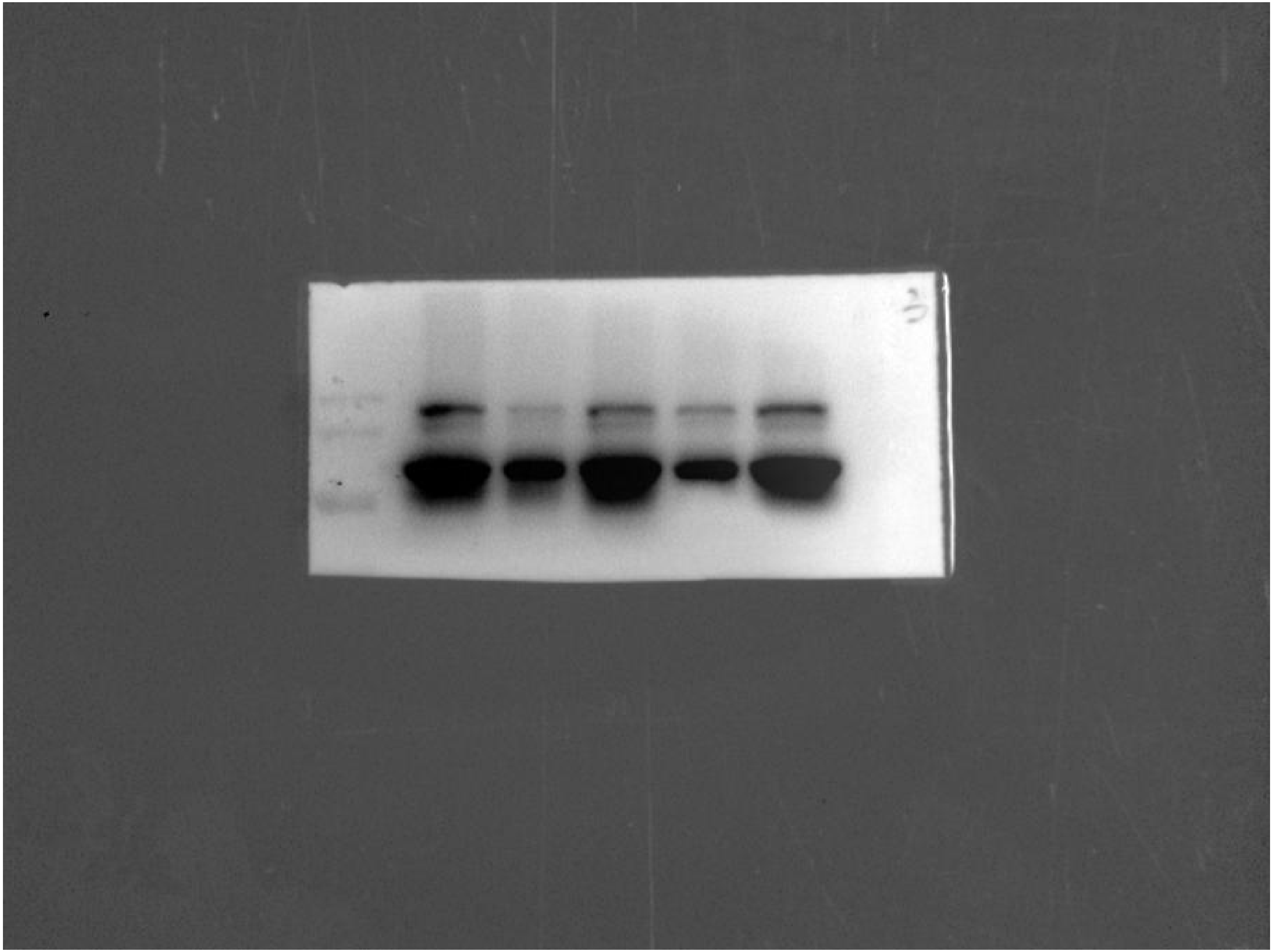

Figure 6A p-VE-cadherin

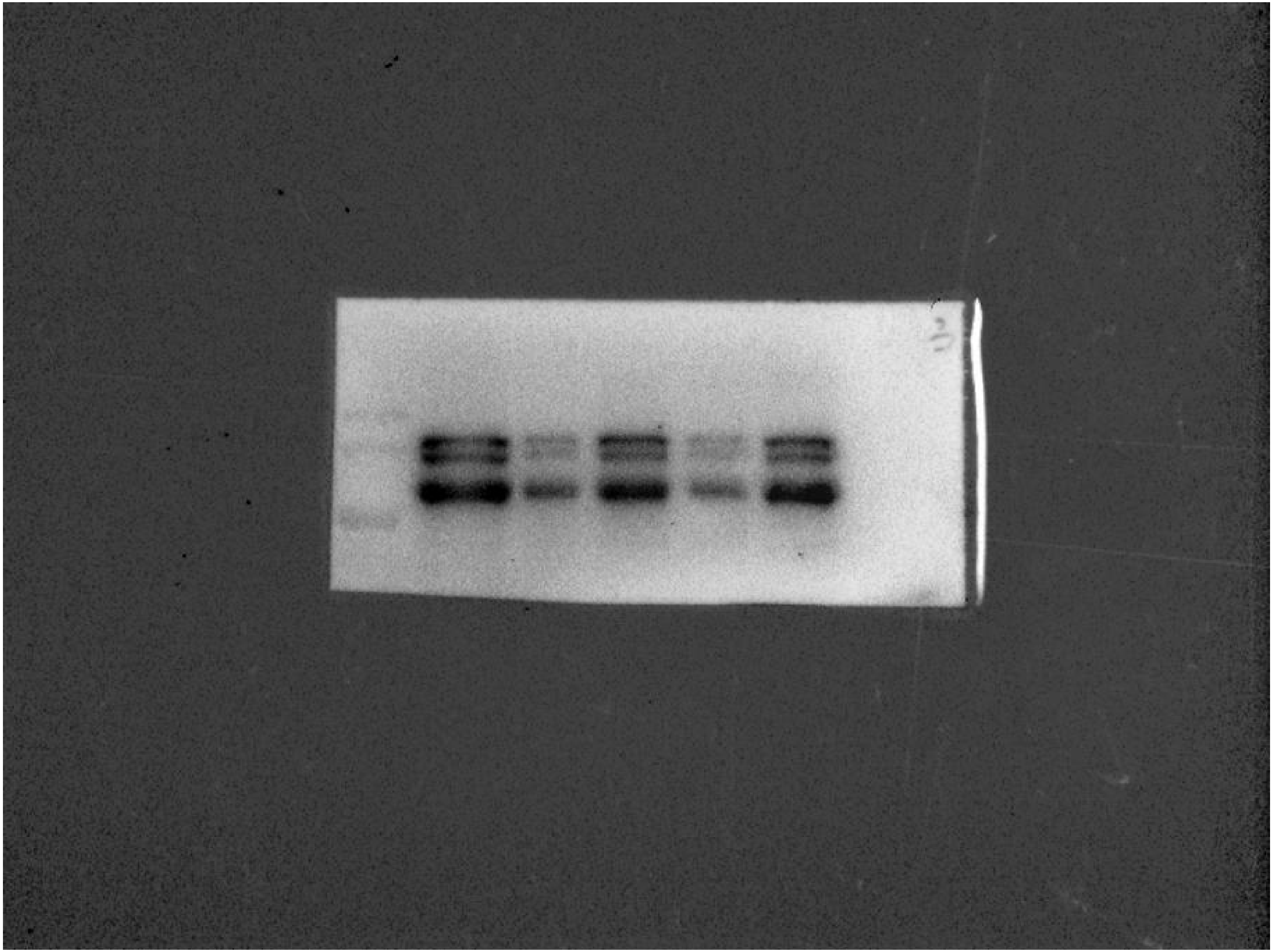

Figure 6A β-catenin

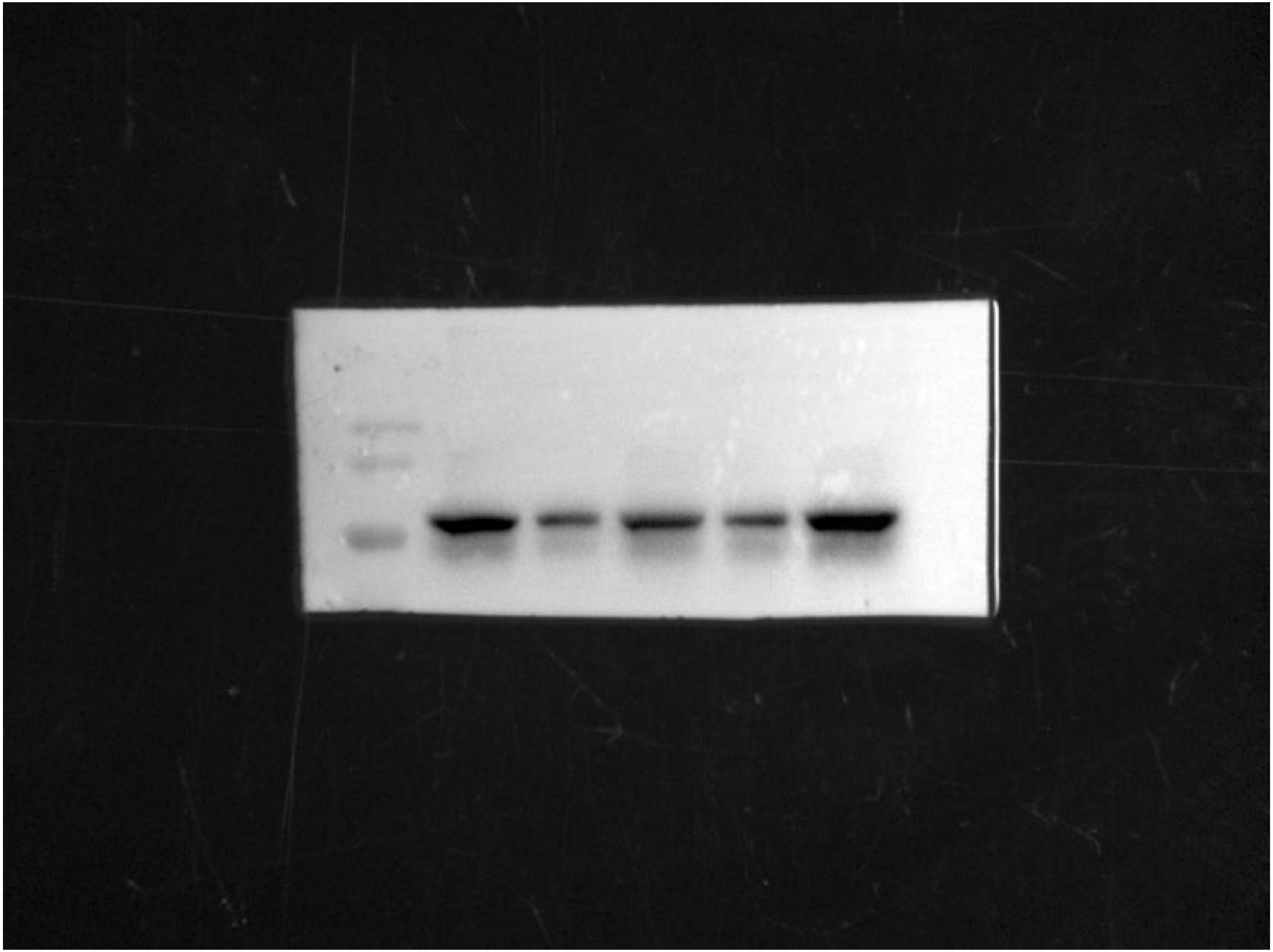

Figure 6A APJ

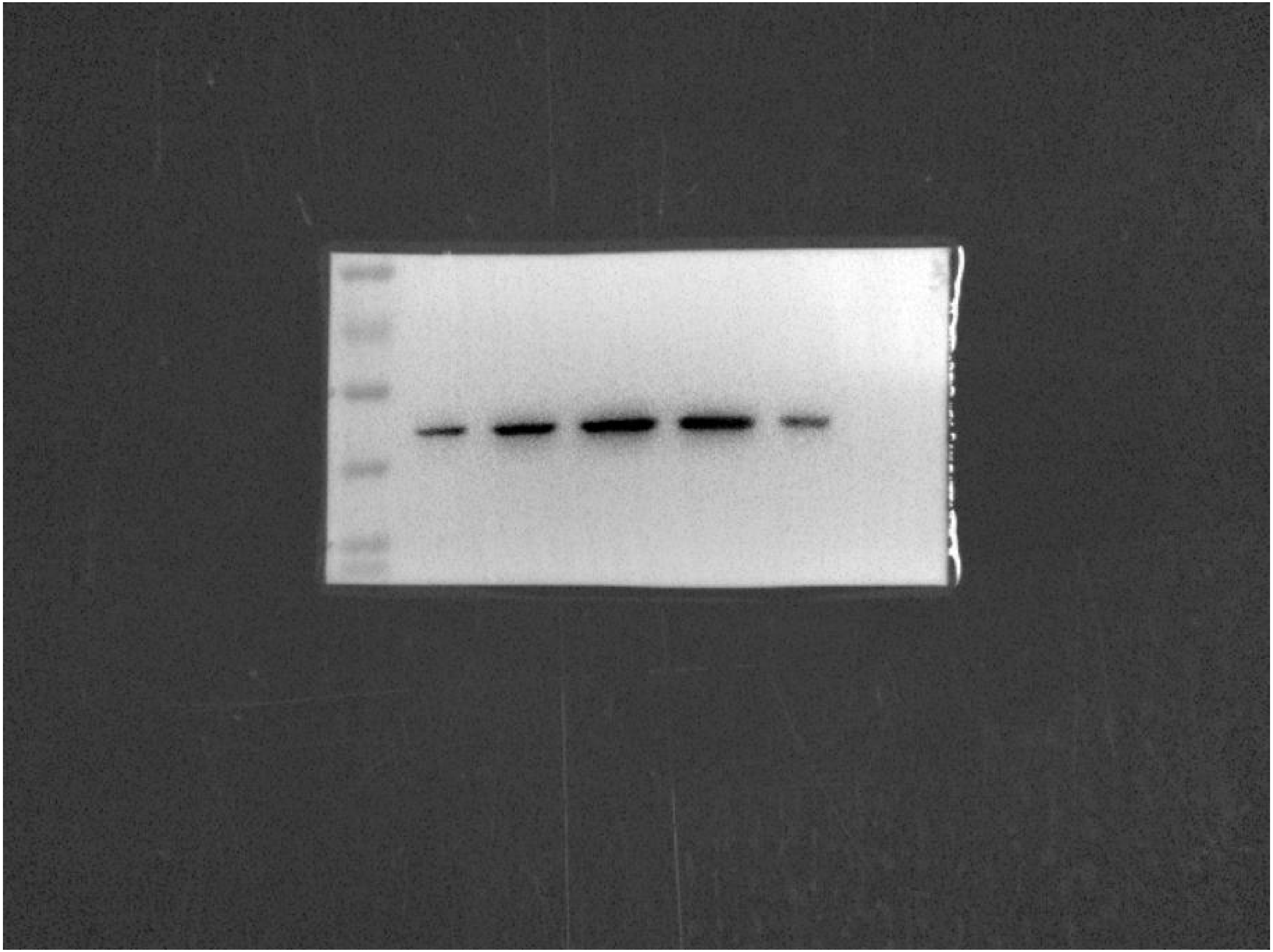

Figure 6A GAPDH

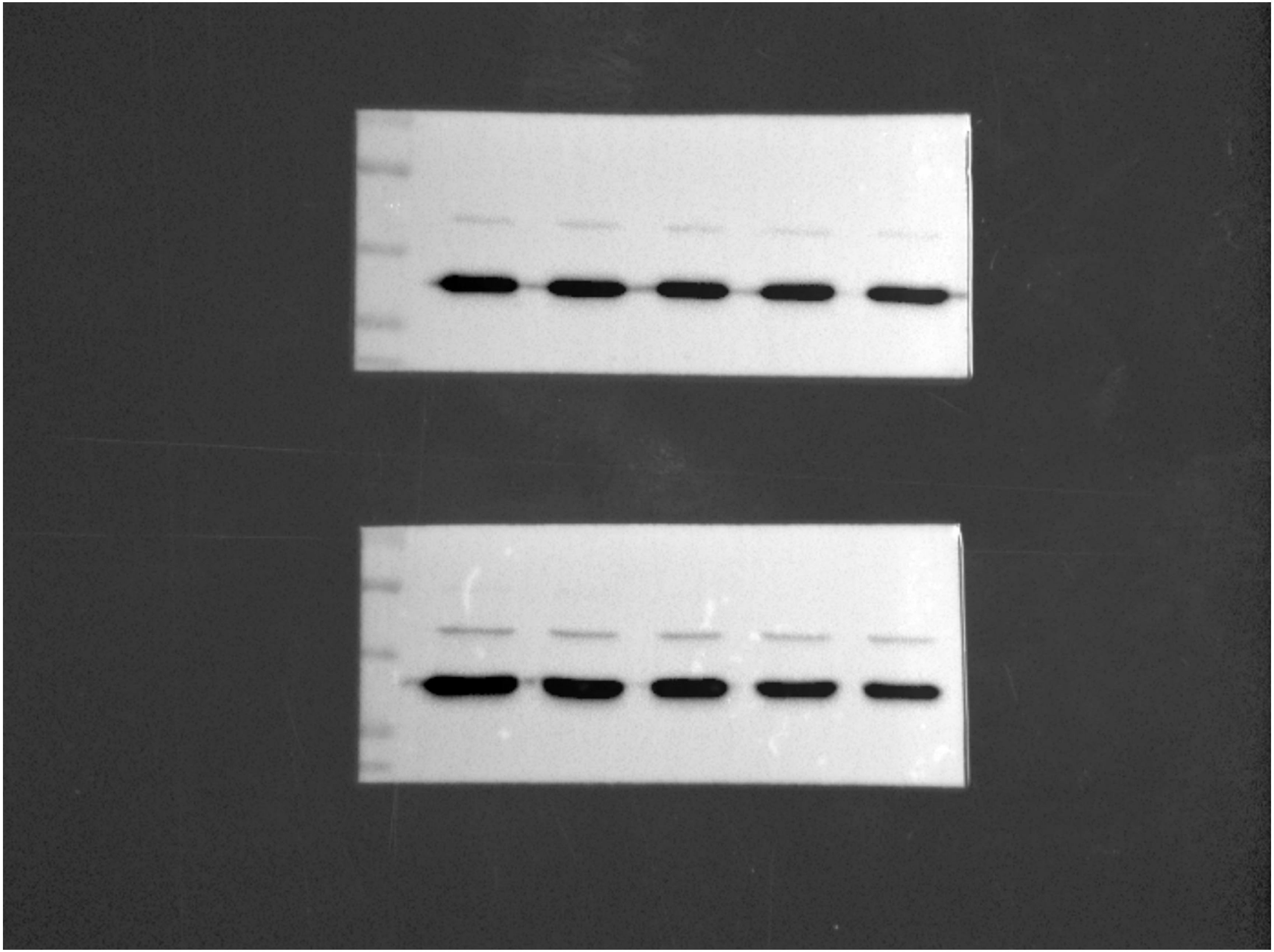

Supplement: Supplementary file 2 — Supplementary Information 2. [file 41598_2023_27889_MOESM2_ESM.pdf]
